# Multiple Quorum Quenching Enzymes Are Active in the Nosocomial Pathogen *Acinetobacter baumannii* ATCC17978

**DOI:** 10.3389/fcimb.2018.00310

**Published:** 2018-09-13

**Authors:** Celia Mayer, Andrea Muras, Manuel Romero, María López, María Tomás, Ana Otero

**Affiliations:** ^1^Department of Microbiology and Parasitology, Faculty of Biology-CIBUS, Universidade de Santiago de Compostela, Santiago de Compostela, Spain; ^2^Department of Microbiology, Complejo Hospitalario Universitario A Coruña-INIBIC, A Coruña Spain

**Keywords:** *Acinetobacter baumannii*, quorum sensing, AHL, quorum quenching, lactonase

## Abstract

*Acinetobacter baumannii* presents a typical *lux*I/*lux*R quorum sensing (QS) system (*abaI*/*abaR*) but the acyl-homoserine lactone (AHL) signal profile and factors controlling the production of QS signals in this species have not been determined yet. A very complex AHL profile was identified for *A. baumannii* ATCC17978 as well as for *A. nosocomialis* M2, but only when cultivated under static conditions, suggesting that surface or cell-to-cell contact is involved in the activation of the QS genes. The analysis of *A. baumanni* clinical isolates revealed a strain-specific AHL profile that was also affected by nutrient availability. The concentration of OHC12-HSL, the major AHL found in *A. baumannii* ATCC17978, peaked upon stationary-phase establishment and decreases steeply afterwards. Quorum quenching (QQ) activity was found in the cell extracts of *A. baumannii* ATCC17978, correlating with the disappearance of the AHLs from the culture media, indicating that AHL concentration may be self-regulated in this pathogen. Since QQ activity was observed in strains in which AidA, a novel α/β-hydrolase recently identified in *A. baumannii*, is not present, we have searched for additional QQ enzymes in *A. baumannii* ATCC17978. Seven putative AHL-lactonase sequences could be identified in the genome and the QQ activity of 3 of them could be confirmed. At least six of these lactonase sequences are also present in all clinical isolates as well as in *A. nosocomialis* M2. Surface-associated motility and biofilm formation could be blocked by the exogenous addition of the wide spectrum QQ enzyme Aii20J. The differential regulation of the QQ enzymes in *A. baumannii* ATCC17978 and the full dependence of important virulence factors on the QS system provides a strong evidence of the importance of the AHL-mediated QS/QQ network in this species.

## Introduction

*Acinetobacter* spp. are Gram-negative, strictly aerobic coccobacilli belonging to the Gammaproteobacteria class and *Pseudomonales* order, broadly distributed in the natural environment, including soil, water and vegetation (Bergogne-Bérézin and Towner, 1996). Although the genus *Acinetobacter* includes non-pathogenic species that are present in the human skin, several *Acinetobacter* species cause a variety of opportunistic nosocomial infections including septicemia, pneumonia, endocarditis, meningitis, skin, wound, and urinary tract infections (Bergogne-Bérézin and Towner, 1996; Towner, 2009). *Acinetobacter baumannii*, the most relevant pathogenic species in the genus, has emerged as one of the most troublesome hospital-acquired pathogens (Peleg et al., 2008). Since the increase in the prevalence of multidrug resistant strains has reduced the treatment options for this pathogen (Rice, 2006; Peleg et al., 2008), *A. baumannii* is considered as an ESKAPE pathogen (Rice, 2008). Therefore, a better understanding of the mechanisms controlling the expression of virulence traits and propagation in *Acinetobacter* spp. has become critical for the discovery and development of new therapeutic strategies for these bacteria.

Important virulence traits such as motility and biofilm formation have been proposed to be under control of an *N-*acyl-homoserine lactone (AHL)-mediated quorum sensing (QS) system in different species of the *A. calcoaceticus-A. baumannii* complex (Niu et al., 2008; Kang and Park, 2010; Clemmer et al., 2011; Anbazhagan et al., 2012; Bhargava et al., 2012; Chow et al., 2014; Oh and Choi, 2015). *A. nosocomialis* M2, formerly identified as *A. baumannii* (Carruthers et al., 2013), presents a typical LuxI/LuxR-type QS network, constituted by the AHL-synthase AbaI and the AHL-receptor and transcriptional activator AbaR (Niu et al., 2008; Bhargava et al., 2010). Genes homologous to *abaI* and *abaR* of *A. nosocomialis* M2 can be found in *A. baumannii* (Smith et al., 2007; Niu et al., 2008) and in the genomes of other *Acinetobacter* species (Kang and Park, 2010; Bitrian et al., 2012; How et al., 2015; Oh and Choi, 2015). Moreover, a number of studies have described the generation of AHL signals in members of the genus (Niu et al., 2008; Chan et al., 2011, 2014; How et al., 2015). In *A. nosocomialis* M2, the signal *N*-hydroxydodecanoyl-L-homoserine lactone (OHC12-HSL) has been identified as the major AHL together with minor amounts of five additional signals (Niu et al., 2008). OHC10-HSL was identified as the major AHL produced when the synthase of a clinical isolate of *A. baumannii* was over-expressed in *Escherichia coli* (Chow et al., 2014), but the profile and factors affecting AHL production in cultures of *A. baumannii* has not been reported yet.

The capacity to degrade AHL-type QS signals, an activity known as Quorum Quenching (QQ) has been described in several environmental *Acinetobacter* isolates: the acylase AmiE, identified in *Acinetobacter* sp. Ooi24, isolated from activated sludge in a wastewater treatment plant (Ochiai et al., 2014) and the lactonase AidE, identified in *Acinetobacter* sp. 77 (Liu et al., 2017). Several other environmental strains with QQ activity have been described, but the enzymes responsible for the activity were not identified (Kang et al., 2004; Chan et al., 2011; Ochiai et al., 2013; Kim et al., 2014; Arivett et al., 2015). Putative lactonases have been identified in *A. baumannii* genomes of environmental and clinical origin (Vallenet et al., 2008; Kang and Park, 2010; Arivett et al., 2015), but the QQ activity of the strains or the catalytic activity of the enzyme has not been demonstrated. Recently, a novel enzyme capable of degrading AHLs has been identified in *A. baumannii* (López et al., 2017). The enzyme, named AidA, is a novel α/β hydrolase and is present in several clinical isolates of *A. baumannii*, but could not be identified in isolate Ab7, the only motile strain under permissive conditions. The role of AHL-mediated QS in motility has been previously described in *A. nosocomialis* M2 (Clemmer et al., 2011) and therefore, the absence of AidA could have explained the increase in motility capacity in this strain (López et al., 2017). This hypothesis was further supported by the fact that the addition of the wide spectrum AHL-degrading enzyme Aii20J (Mayer et al., 2015) completely blocked motility in Ab7 (López et al., 2017). Nevertheless, an analysis of the genomes of the well-studied strain *A. baumannii* ATCC17978, that is motile under permissive conditions (unpublished results), revealed that AidA is present, opening a question on the role of AidA in the control of QS-related phenotypes.

Therefore, in this work we have analyzed the production of QS signals and QQ capacity in *A. baumannii* ATCC17978 and compare it with the well-studied species *A. nosocomialis* M2 (formerly classified as *A. baumannii*). AHL production and QQ activity was also studied in 7 clinical isolates of *A. baumannii* (López et al., 2017). Since the analysis revealed the presence of QQ activity in *A. nosocomialis* M2, but the QQ enzyme AidA is not present in the genome of this strain, we also carried out a search in order to identify possible additional QQ enzymes in *A. baumannii* ATCC17978. We provide evidence that growth under static conditions is critical for AHL production in these pathogens and that QQ activity could be responsible for the decrease of AHL concentration observed in the onset of stationary phase. Three AHL-lactonases could be cloned and over-expressed in *E. coli*, confirming its QQ activity. The exogenous addition of the wide-spectrum QQ enzyme Aii20J (Mayer et al., 2015) completely blocked surface-associated motility and biofilm formation in *A. baumannii* ATCC17978, confirming the relevance of the QS system for key virulence traits in this species.

## Materials and methods

### Bacterial strains, culture conditions, and genetic methods

Bacterial strains, plasmids, and primers used in this study are listed in Table 1. LB broth and agar were used to routinely grow and maintain *Acinetobacter* spp. at 37°C. *Chromobacterium violaceum* biosensor strains were routinely cultured on LB medium at 30°C. Antibiotics were added at final concentrations of 25–50 μg/mL kanamycin or 25 μg/mL tetracycline as required.

**Table 1:** Bacterial strains, plasmids, and primers used in this study.

**Strain or plasmid**	**Description**	**Source or references**
**STRAINS**		
***Acinetobacter baumannii***		
ATCC17978		ATCC^a^
Ab1 (ROC013)	*A. baumannii* clinical isolate (respiratory). TM: ST2	INIBIC^b^
Ab2 (COR005)	*A. baumannii* clinical isolate (ulcer). TM: ST186	INIBIC^b^
Ab3 (PON002)	*A. baumannii* clinical isolate (respiratory). TM: ST52	INIBIC^b^
Ab4 (VAL001)	*A. baumannii* clinical isolate (respiratory). TM: ST169	INIBIC^b^
Ab5 (DOM009)	*A. baumannii* clinical isolate (respiratory). TM: ST80	INIBIC^b^
Ab6 (HMV001)	*A. baumannii* clinical isolate (exudate). TM: ST181	INIBIC^b^
Ab7 (HUI001)	*A. baumannii* clinical isolate (blood). TM: ST79	INIBIC^b^
***Acinetobacter nosocomialis***		
M2		Niu et al., 2008
***Chromobacterium violaceum***		
CV026	AHL biosensor, Km^r^	McClean et al., 1997
VIR07	AHL biosensor, Km^r^	Morohoshi et al., 2008
***Escherichia coli***		
BL21(DE3)plysS	F– *omp*T *hsd*S_B_ (r_B_-, m_B_-) *dcm gal* λ(DE3) pLysS Cm^r^	Promega
XL1blue	*recA1 endA1 gyrA96 thi-1 hsdR17 supE44 relA1 lac* [*F′ proAB lacIq ZΔM15* Tn10 (Tet^r^)]	Agilent
**PLASMIDS**		
pET28c(+)	Cloning vector, Km^r^	Novagen
pET28c(+)-*aidA*	pET28c(+) containing *aidA* gene from *A. baumannii* ATCC17978	This study
pET28c(+)-*aii20J*	pET28c(+) containing *aii20J* gene from *Tenacibaculum* sp. 20J	Mayer et al., 2015
pET28c(+)-A1S_0383	pET28c(+) containing *a1s_0383* gene from *A. baumannii* ATCC17978	This study
pET28c(+)-A1S_1876	pET28c(+) containing *a1s_1876* gene from *A. baumannii* ATCC17978	This study
pET28c(+)-A1S_2662	pET28c(+) containing *a1s_2662* gene from *A. baumannii* ATCC17978	This study
**Primers**	**Sequence (5**′**-3**′**)**	**Use in this work**
*abaI*_Fwd	TGTGCCAGACTACTACCCAC	qRT-PCR
*abaI*_Rev	TGCTAGAGGAAGGCGGATTT	qRT-PCR
*abaR*_Fwd	TTGGTCGAGTCAATCTGCAA	qRT-PCR (Eijkelkamp et al., 2013)
*abaR*_Rev	CTCGGGTCCCAATAAAATCA	qRT-PCR (Eijkelkamp et al., 2013)
*csuD*_Fwd	AGTCACAACATCGGTCCCAT	qRT-PCR
*csuD*_Rev	AAGTTCGGTGCGTCCTTCTA	qRT-PCR
*rpoB*_Fwd	GTGCTGACTTGACGCGTGAT	qRT-PCR (Park and Ko, 2015)
*rpoB*_Rev	AGCGTTCAGAAGAGAAGAACAAGTT	qRT-PCR (Park and Ko, 2015)
A1S_0383_Fwd	ACCAGGTCACGTCATGTTCT	qRT-PCR
A1S_0383_Rev	TGGTACTCATTGGCCCATGT	qRT-PCR
A1S_1708_Fwd	ATTGAAGCGCGTTACACACC	qRT-PCR
A1S_1708_Rev	ATAGTGTTGTCAGGCAGGCT	qRT-PCR
A1S_1876_Fwd	GCAGTCATATGGTCCGCATG	qRT-PCR
A1S_1876_Rev	TTAGCAACCCGTCAATGTGC	qRT-PCR
A1S_2194_Fwd	TCCCTGGCATTACTCATCCC	qRT-PCR
A1S_2194_Rev	TTCAAATAGTCGCCCGCATC	qRT-PCR
A1S_2662_Fwd	TCTGCTTCACGTTCATGAGC	qRT-PCR
A1S_2662_Rev	CTGCGAGTTGTTTTGGTCCA	qRT-PCR
A1S_2864_Fwd	GCCACTGAATACAATGCTGC	qRT-PCR
A1S_2864_Rev	TCGCAATACCACAATGTCCG	qRT-PCR
*aidA*_Fwd	TCGCTGCACGTTTTGTACTC	qRT-PCR
*aidA*_Rev	CCATCGGCGTAGTGCTTAAT	qRT-PCR
0383Fwd	GATTAACCATGGTACTGCAAGTCAAAATTGTTCCAG (NcoI)^c^	Cloning
0383Rev	GCTATGAATTCAAACCCGCTTTACCTGCGACAAACG (EcoRI)^c^	Cloning
1876Fwd	GATTAACCATGGTACAACAACCTCTAGTAAAAGA(NcoI)^c^	Cloning
1876Rev	GCTATGAATTCAAAAAGTAATTAAATGGGATT (EcoRI)^c^	Cloning
2662Fwd	GATTAACCATGGTAAAAAAACTATTTGTAGCCTTAGG (NcoI)^c^	Cloning
2662Rev	GCTATGAATTCAATTTATCAAGACTGTTATTA (EcoRI)^c^	Cloning
*aidA*Fwd	GATTAACCATGGTA.GGTAAAAGTCTAAATAATGT	Cloning
*aidA*Rev	GCTATGAATTCAACTTGACTGGAACGATGCGTTTA	Cloning
T7Fwd	TAATACGACTCACTATAGGGGAA	Universal primer
T7Rev	GCTAGTTATTGCTCAGCGG	Universal primer
*luxI* PF	GGTTGGGAGTTGAACTGTCC	*abaI* amplification (Bitrian et al., 2012)
*luxI* PR	GGTTGGGAGTTGAACTGTCC	*abaI* amplification (Bitrian et al., 2012)
*luxR* PF	TCGGATTTGATTATTGCGCTTATG	*abaI* amplification (Bitrian et al., 2012)
*luxR* PR	ACAGCTCGAATAGCTGCTG	*abaI* amplification (Bitrian et al., 2012)

a *American Type Culture Collection*.

b *Instituto de Investigación Biomédica (A Coruña)*.

c *Restriction sites for indicated enzymes are underlined*.

### AHL profile identification

The AHL profiles of *A. baumannii* ATCC17978 and *A. nosocomialis* M2 were obtained from 100 mL static and shaken liquid cultures grown for 0, 6, 12, 17, 24, 36, and 48 h at 37°C in LB. To compare the effect of culture media static and shaken cultures of *A. baumannii* ATCC17978 were also grown in LB (1% NaCl, 1% tryptone, and 0.5% yeast extract), low-nutrient low-salt LB (0.5% NaCl, 0.2% tryptone, and 0.1% yeast extract; LNLS-LB), low-salt LB (0.5% NaCl, 1% tryptone, and 0.5% yeast extract; LS-LB), or buffered LB (PIPES buffer, 200 mM, pH 6.7) for 17 h at 37°C. Cells were removed by centrifugation and supernatants were extracted twice with an equal volume of dichloromethane. Solvent was evaporated to dryness in a rotary evaporator at 40°C. Extracts were then dissolved in 1 mL of acetonitrile and signals were identified and quantified by HPLC-MS methodology using AHL synthetic standards as reference (Romero et al., 2014). Extracts from non-inoculated culture media incubated the same way were used as controls.

### Detection of quorum quenching activity

*C. violaceum*-based solid plate assays were carried out to detect AHL degradation activity in *A. baumannii* ATCC17978 and *A. nosocomialis* M2 as described before (Romero et al., 2010). In brief, *Acinetobacter* spp. pellets were collected from LB cultures at different times of the same growth curve of the previous section (6, 12, 17, 24, 36, and 48 h), washed in phosphate buffer saline (PBS) pH 6.7, disrupted by sonication on ice, centrifuged and filtered (0.20 μm) to obtain the cell extracts. Five hundred microliters of aliquots from each cell extract were exposed to 10 μM C6 or C12-HSL and incubated for 24 h at 22°C with shaking. In order to detect AHL inactivation activity, 100 μL of the reaction mixtures were spotted in wells made in LB plates overlaid with 5 mL of a 1/100 dilution of an overnight culture of *C. violaceum* CV026 for C6-HSL or VIR07 for C12-HSL in soft agar (0.8%). Plates were incubated for 24 h at 30°C, and the production of violacein was examined. PBS buffer plus AHLs incubated the same way were used as controls in all plates.

Confirmation of the QQ activity of *A. baumannii* ATCC17978 was performed by HPLC-MS analysis. The cell extract from a 50 mL culture in LB of ATCC17978 grown for 24 h was obtained. Then, C12 and OHC12-HSL signals (10 μM) were incubated with the cell extract at 22°C with shaking. After 24 h exposure, 200 μL of the reaction mixtures were extracted three times with the same volume of ethyl acetate with or without previous acidification to pH 2.0 for 24 h. Solvent was evaporated under nitrogen flux and suspended in acetonitrile for AHL quantification as previously described (Romero et al., 2014). PBS plus the same amount of C12 or OHC12-HSL were used as controls.

### Identification and cloning of QQ sequences

The genomic DNA from different clinical isolates and *A. baumannii* ATCC17978 was used as template for PCR detection of *abaI/abaR* homologous. Genomic DNA was extracted with Wizard® Genomic DNA Purification Kit (Promega). Primers *luxI* PF, *luxI* PR, *luxR* PF, and *luxR* PR were used for *abaI/abaR* homologous amplification with the PCR conditions used by Bitrian et al. (2012). PCR products of the synthase and receptor (about 370 and 600 bp, respectively) were then sequenced, and analyzed using the MEGA 6 phylogenetic tool software package (Tamura et al., 2013) using the default parameters.

Bioinformatic tools such as blast (https://blast.ncbi.nlm.nih.gov/Blast.cgi) and cd-search (https://www.ncbi.nlm.nih.gov/Structure/cdd/wrpsb.cgi) from NCBI were used for identification of new QQ sequences in the *A. baumannii* ATCC17978 genome. Sequences were aligned with Clustal Omega (https://www.ebi.ac.uk/Tools/msa/clustalo/) or MUSCLE programs from EMBL-EMI (https://www.ebi.ac.uk/Tools/msa/muscle/) and shaded using the GeneDoc 2.7 program.

QQ sequences were amplified by PCR using genomic DNA and primers listed in Table 1. PCR conditions included denaturation at 94°C, 5min; 30 cycles of 95°C, 45 s; 55°C, 45 s; and 72°C, 1 min, with a final extension for 10 min. The PCR products from QQ enzymes were purified, digested with EcoRI and NcoI (Thermo Scientific), and cloned into the EcoRI and NcoI sites of vector pET28c(+) using T4DNA ligase (Thermo Scientific), to introduce six histidine residues in the C terminus of the protein, and transformed by electroporation into *E. coli* XL1blue and then in *E. coli* BL21(DE3) plysS. The *E. coli* BL21(DE3)plysS strains expressing recombinant proteins were inoculated into fresh LB medium with kanamycin (25 μg/mL) at 37°C with shaking. After the OD600 of the culture reached 0.6, the protein expression was induced by the addition of isopropyl-D-thiogalatopyranoside (IPTG) to a final concentration of 1 mM followed by further incubation for 5 h. After incubation, cells were harvested by centrifugation, resuspended with 20 mL of PBS buffer, lysed by sonication on ice, and centrifuged at 4°C (2,000 × g for 5 min). QQ enzymes were purified using the His GraviTrap™ affinity column (GE Healthcare) protein purification kit. Purified proteins were measured by a UV-Vis Spectrophotometer Q5000 (Quawell) and analyzed with 12% SDS-PAGE.

### Characterization of QQ enzymes

QQ activity of QQ purified enzymes was confirmed with *C. violaceum* based assays. Purified proteins concentration was measured and the minimum active concentration (MAC) of each enzyme was stablished as the protein concentration in the highest decimal dilution being able to completely remove the activity of a 10 μM solution of C12-HSL in 24 hours, as detected by the *C. violaceum* VIR07 biosensor assay.

In order to determinate the specificity of purified QQ proteins a 10xMAC concentration of each enzyme was mixed with several AHLs at 10 μM in PBS pH 6.7, for 24 h, and incubated at 22°C, with shaking. The remaining signal was detected in solid plate assay with *C. violaceum* CV026 or VIR07 as explained before. Controls of PBS with the same amount of AHL were processed in the same way. AHL degradation specificity of purified enzymes was evaluated with synthetic signals: *N*-hexanoyl-L-homoserine lactone (C6-HSL), *N*-octanoyl-L-homoserine lactone (C8-HSL), *N*-decanoyl-L-homoserine lactone (C10-HSL), *N*-3-oxodecanoyl-homoserine lactone (OC10-HSL), *N*-hydroxydecanoyl-L- homoserine lactone (OHC10-HSL), *N*-dodecanoyl-L- homoserine lactone (C12-HSL), *N*-3-oxododecanoyl-L- homoserine lactone (OC12-HSL), *N*-hydroxydodecanoyl-L- homoserine lactone (OHC12-HSL), *N*-3-oxotridecanoyl-L- homoserine lactone (OC13-HSL), *N*-tetradecanoyl-L-homoserine lactone (C14-HSL), and *N*-3-oxotetradecanoyl-L-homoserine lactone (OC14-HSL).

### Bacterial RNA isolation and quantitative real time PCR (qPCR)

For relative transcript levels quantification of selected genes, quantitative PCR (qPCR) was performed using cDNA from cultures of *A. baumannii* ATCC17978 grown at 37°C in LB or LS-LB with or without agitation. *Acinetobacter baumannii* cells were grown up to an optical density (600 nm) of 0.6 and total RNA was isolated using the RNase Mini Kit (Qiagen) and then treated with the Turbo DNA-free DNase kit (Ambion) following manufacture's instructions. DNA contamination was evaluated by PCR with 1 μL of purified RNA as template. RevertAid Reverse Transcriptase and random hexamers (Thermo Fisher Scientific) were used to synthesize complementary deoxyribonucleic acid (cDNA) according to the manufacturer's protocol.

qPCR was performed by using FastStart SYBR Green Master (Roche). Each 20 μL reaction mixture contained 1X FastStart SYBR Green Master, 300 nM primers, and 2 ng cDNA template. The oligonucleotides used in this study for qPCR were designed using the software Primer3 (http://bioinfo.ut.ee/primer3/) and are listed in Table 1. The efficiency of each primer pair was determined by carrying out RT-PCR on serial dilutions of cDNA, and the specificity was verified by melting-curve analyses (1 cycle of 95°C for 1 min and another cycle of 60°C for 1 min followed by melting at 0.5°C increments for 10 s to 95°C). Following the verification of primer efficiency and specificity, qPCR analyses were routinely carried out with an iCycler iQ5 real-time PCR detection system (Bio-Rad) according to the following amplification protocol: 95°C for 10 min followed by 40 cycles of 95°C for 20 s, 56°C for 30 s, and 72°C or 40 s. qPCRs were performed in duplicate and samples containing no reverse transcriptase or template RNA were included as negative controls. Data were analyzed by using iQ5 Optical System software (Bio-Rad), and the relative quantification was determined by the ΔΔ*C*_*T*_ method normalizing to the transcription levels of the housekeeping *rpoB* gene (Livak and Schmittgen, 2001).

### Motility and biofilm assays

Surface-associated motility assays were performed on Petri dishes with LB or LNLS-LB in 0.25% Eiken Agar (Eiken Chemical Co. Ltd. Japan). One microliter of 17-h cultures at an OD600 of 0.3 was inoculated in the center of the plates. The QQ enzyme Aii20J was mixed with the inoculum at a concentration of 20 μg/mL (López et al., 2017). Plates were incubated at 37°C for 14 h. Three plates were inoculated for each condition and experiments were repeated at least twice.

Biofilm was formed on the surface of suspended 18x18 mm coverslips using a modification of the Amsterdam active attachment model (Exterkate et al., 2010). Coverslips were submerged vertically in 3 mL of low-salt (0.5%) LB medium in 12-well cell culture plates inoculated with 17 h cultures at an OD600 of 0.05. Cultures were maintained for 4 days at 37°C and culture medium was exchanged daily. The QQ enzyme Aii20J (Mayer et al., 2015) was added with every medium exchange at a concentration of 20 μg/mL. For each condition two coverslips were stained with crystal-violet (Muras et al., 2018b) and another two were stained with LIVE/DEAD® BacLight™ Bacterial Viability Kit (Invitrogen) and observed with a Leica TCS SP5 X confocal laser scanning microscope (Leica Microsystem Heidelberg GmbH, Mannheim, Germany) with an HC PL APO 10 × /0.4 CS objective.

### Statistical methods

Student's *t*-test for independent samples (*P* < 0.05) were applied for all statistical analyses.

## Results

### Identification of AHL profile in *A. baumannii* ATCC17978

We first analyzed the AHL profile and production kinetics in *A. baumannii* ATCC17978 by sampling at different time points during the growth curve. The cultures were done in LB and LB buffered with PIPES (pH 6.5) in order to avoid the spontaneous hydrolysis of the AHLs as basic pH (Yates et al., 2002). The same experiment was carried out with the well-studied species *A. nosocomialis* M2 in order to compare the production kinetics between both strains. Surprisingly, no AHL signal could be detected in 100-fold-concentrated extracts of culture media supernatants in shaken cultures for both, *A. baumannii* ATCC17978 and *A. nosocomialis* M2, as well as in the other *A. baumannii* clinical isolates analyzed (data not shown). On the contrary, a complex AHL profile was detected when the strains were grown under static conditions (Tables S1, S2). The differences in growth under static and shaken conditions were small after the first 10 h of culture, and therefore oxygen limitation can be disregarded as the main factor affecting AHL production under static conditions (Figure S1).

**Figure 1:**
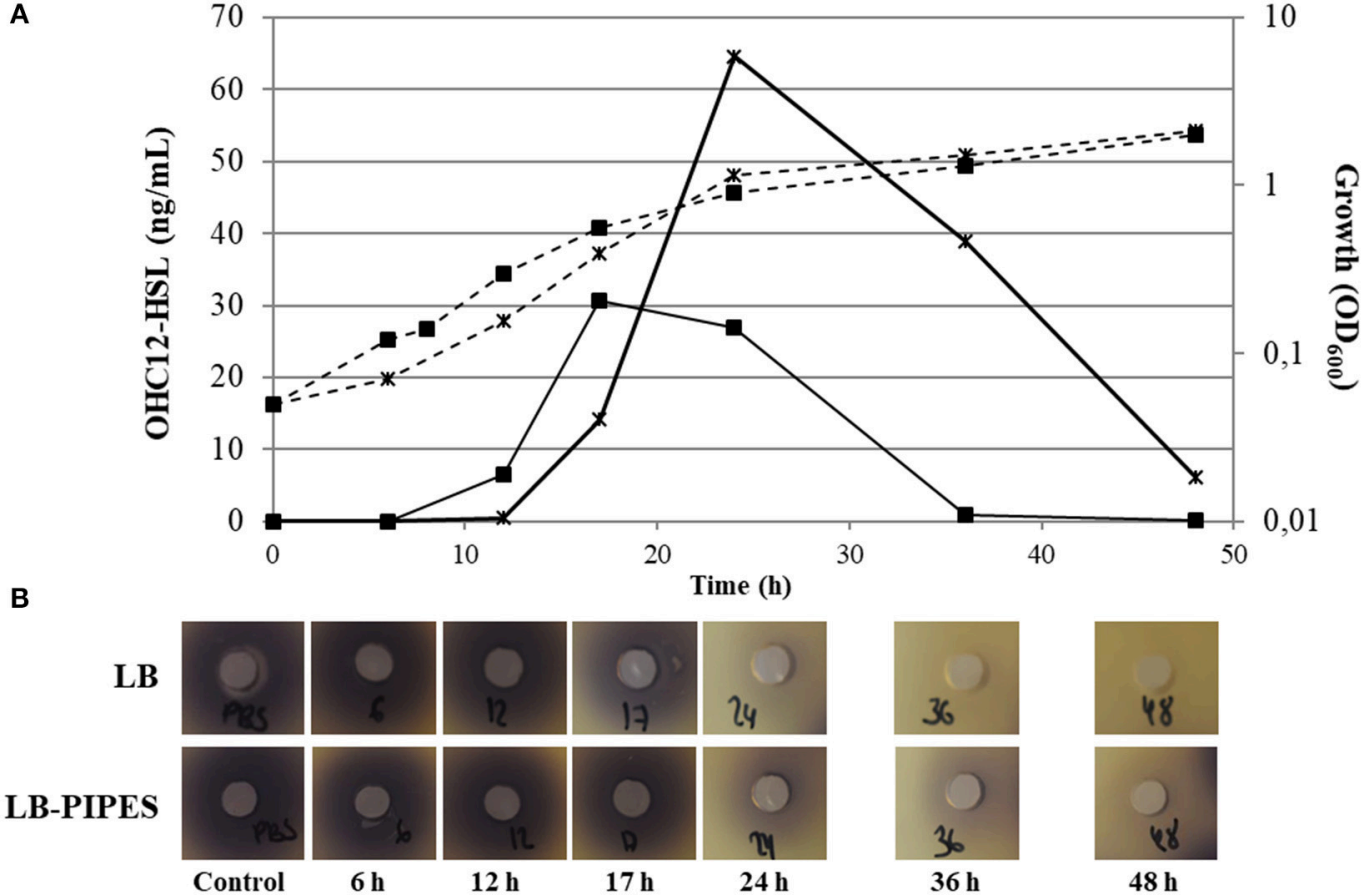
**(A)** OHC12-HSL production kinetics (continuous lines) and growth curves (discontinuous lines) in static cultures of *A. baumannii* ATCC17978 grown in LB (filled squares) or buffered LB (black cross) (200 mM PIPES buffer, pH 6.7) at 37°C for 48h. **(B)** Bioassay with *C. violaceum* VIR07 to detect QQ activity in cell extracts of *A. baumannii* ATCC17978. The degradation activity against exogenous C12-HSL (10 μM) was assayed in cell extracts obtained at different time points of the growth curve (6, 12, 17, 24, 36, and 48 h) under static conditions. The presence of QQ activity is revealed by the absence of violacein around the wells. PBS plus AHL samples were treated in the same way and were used as negative controls (Control).

OHC12-HSL was identified as the major AHL signal found in the culture medium of *A. baumannii* ATCC17978 static cultures (Figure S2, Table S1). This AHL was also the main signal detected in *A. nosocomialis* M2, as reported previously (Table S1; Niu et al., 2008). Several additional signals were identified in both species, including OHC10-HSL, OC12-HSL and OHC14-HSL, although at much lower concentrations (Table S1). Minor amounts (0.2–1.5 nM) of C6, OC6, and C8, were also present in *A. baumannii* ATCC17978 only in LB medium in the 17 h sample (data not shown). Besides these 3 short-chain AHLs, OC8, and OHC8 were also found at concentrations lower than 1 nM in *A. nosocomialis* M2 in the same conditions. The concentration of AHLs peaked around 17–24 h of culture, coinciding with late logarithmic phase/early stationary phase, and suffered a steep decrease thereafter, being almost undetectable in supernatant samples after 36 h in unbuffered cultures (Figure 1A). Buffering of the culture media produced a higher maximal concentration of OHC12-HSL that reached 64.56 ng mL^−1^ while only 30.74 ng mL^−1^ were achieved in the unbuffered medium (Figure 1A). In any case, buffering the culture medium did not prevent the decrease in the signal concentration, suggesting an active AHL degradation. Furthermore, as for OHC12-HSL, the concentration of the other AHLs peaked around 17–24 h and decreased thereafter (Table S1). The production kinetics of OHC12-HSL was very similar in *A. nosocomialis* M2 in LB medium, but a lower maximal concentration was achieved: 17.23 ng mL^−1^. Surprisingly, buffering the culture medium almost completely abolished the production of AHLs in this species (Figure S3).

**Figure 2:**
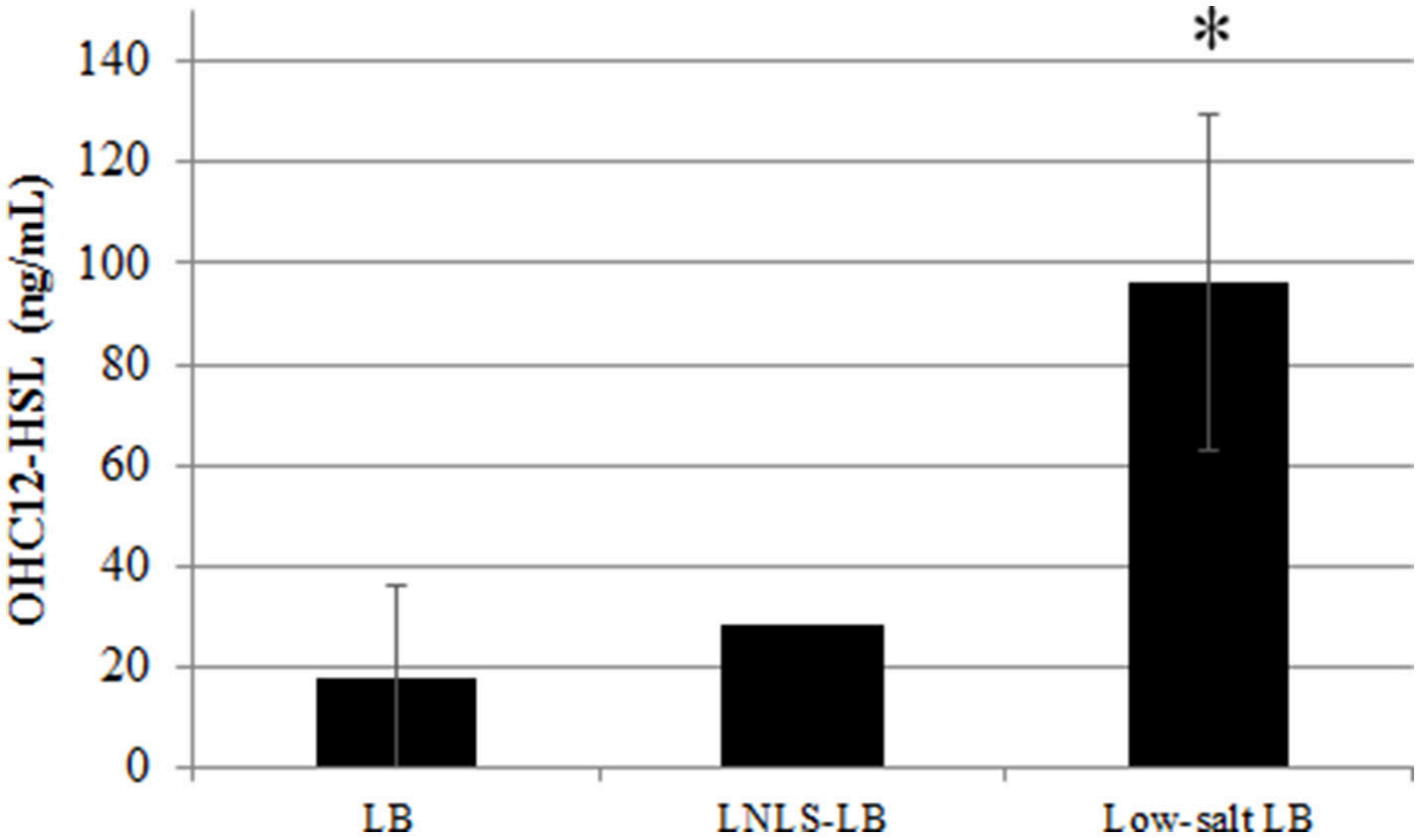
OHC12-HSL concentration of *A. baumannii* ATCC17978 grown in LB, low-nutrient low-salt LB (LNLS-LB), and low-salt LB in static cultures maintained at 37°C during 17 h. Data are means ± SD of three independent experiments. Asterisk indicate statistically significant changes (Student's *t*-test, *p* < 0.05) with respect to the LB control.

To assess the possible effect of culture media on AHL production, AHL concentration was quantified after 17 h in liquid cultures of ATCC17978 grown in LB, nutrient-depleted, low-salt LB (LNLS-LB), and low-salt LB (LS-LB) with or without shaking (Figure 2). Previous results indicated that the expression of QS-related phenotypes, such as motility or biofilm formation, was enhanced with low-salt media (Pour et al., 2011; McQueary et al., 2012) and therefore we intended to see if these phenotypic changes were accompanied by an increase in AHL concentration. Supporting the importance of the static conditions for AHL production, no signal could be detected in supernatants of shaken cultures in any of the culture media tested (data not shown). In contrast, the major signal OHC12-HSL was produced in static cultures with a remarkable increase of concentration in LS-LB medium (Figure 2), a condition that enhances QS-related traits such as motility and biofilm formation (Pour et al., 2011; McQueary et al., 2012). The reduction of NaCl concentration did not affect growth in ATCC17978 (Figure S1).

As for ATCC17978 and *A. nosocomialis* M2, no AHL could be detected in the supernatants of shaken cultures of different *A. baumannii* clinical isolates. In static cultures the AHL profile changed among the clinical isolates analyzed. OHC12-HSL could be detected in only 3 of the 7 strains analyzed after 24 h in LB. This signal could be detected in all strains in LNLS-LB, although at much lower concentration. OHC10-HSL, OC12-HSL, OC14-HSL, and OHC14-HSL were also present in the clinical strains, but with a variable pattern (Table S2). The gene *abaI* could be amplified by PCR in all strains, sharing more than 98% of identity with the *abaI* gene of *A. baumannii* ATCC17978 (Figure S4). *abaR* genes could also be amplified by PCR in all clinical strains, sharing a 99% of identity with the *abaR* gene of ATCC17978 (data not shown).

### Expression of *abaI/abaR* under different conditions

**Figure 3:**
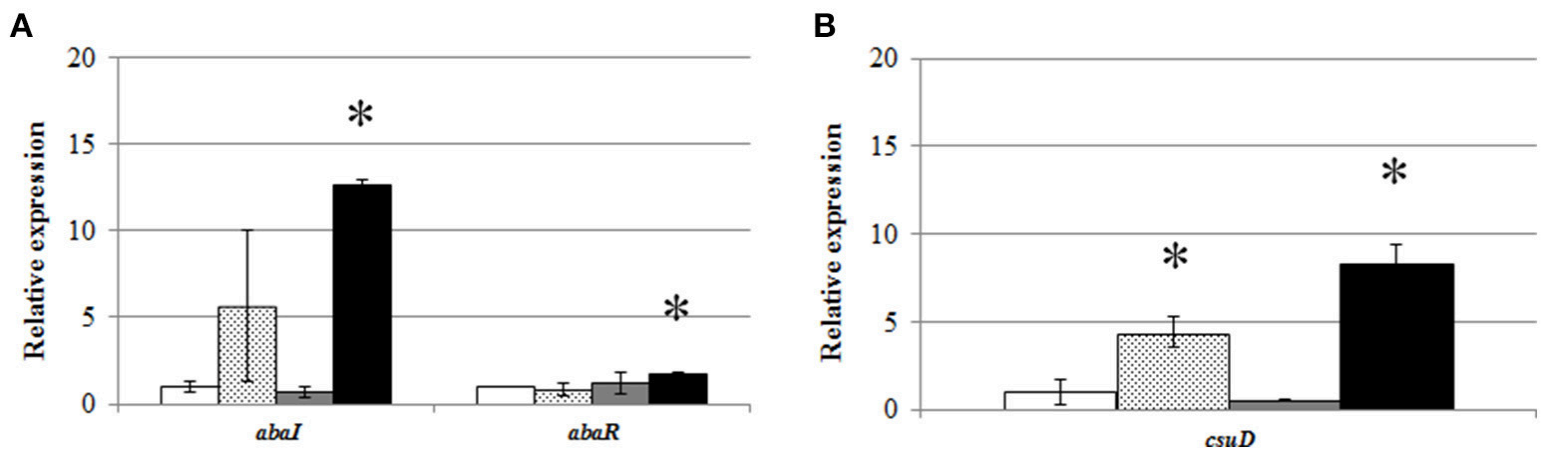
Relative expression of **(A)** *abaI* and *abaR*, and **(B)** *csuD*, genes from *A. baumannii* ATCC17978 in shaken LB (white bars), static LB (dotted bars), shaken low-salt LB (gray bars), or static low-salt LB (black bars) cultures. Gene expression was normalized in relation to the *rpoB* gene and gene expression values in static LB were adjusted to 1.0. Error bars represent the standard deviations. Asterisk indicate statistically significant changes (Student's *t*-test, *p* < 0.05) with respect to the static LB condition.

To assess whether the presence of AHLs only under static conditions was derived from differences in the expression of QS genes, a qPCR was performed with RNA extracted after 6 h from static and shaken cultures of *A. baumannii* ATCC17978 in LB or LS-LB media. Results showed that static cultures and especially static cultures in LS-LB induced the expression of the AHL synthase gene *abaI* (A1S_0109) (Figure 3A), correlating with the presence of AHLs in the culture media. On the contrary, the expression of the synthase in shaken cultures was very low, independently of the culture media used. These results support a correlation between AHL production in ATCC17978 and growth in static conditions, excluding that the absence of AHL production observed in shaken cultures is derived from a fast turn-over of the signals. In contrast, the expression of the AHL receptor *abaR* did not show significant changes except for a slight increase in static cultures with LS-LB medium (Figure 3A). The *csuD* gene, an outer membrane protein required for type I pili biogenesis that is under the control of QS in *Acinetobacter* spp. (Tomaras et al., 2003, 2008; Clemmer et al., 2011; Luo et al., 2015; Chen et al., 2017), showed an expression pattern similar to the QS synthase (Figure 3B), which confirms that static conditions result in the overexpression of the QS operon and their related genes.

### Quorum quenching activity in *A. baumannii* ATCC17978 and *A. nosocomialis* M2

On the basis of the recent identification of the novel α/β hydrolase AidA in several clinical isolates of *A. baumanni* (López et al., 2017), that is also present in the genome of *A. baumannii* ATCC17978, we hypothesized that an enzymatic degradation of AHLs could cause the steep drop of OHC12-HSL concentration observed in static cultures (Figure 1A). To test this, QQ assays against the signals *N*-hexanoyl-L-homoserine lactone (C6-HSL) and *N*-dodecanoyl-L-homoserine lactone (C12-HSL) were performed using cell extracts obtained at different points of the growth curve under static conditions. QQ activity against C12-HSL was detected in cell extracts of *A. baumannii* ATCC17978 in 24-h cultures in both LB and buffered LB, showing a clear correlation with the disappearance of the AHLs (Figure 1B). In ATCC17978 no QQ activity could be found in earlier samples even in 50-fold concentrated cell extracts (data not shown). Interestingly, none of the cell extracts was able to degrade C6-HSL, suggesting that the QQ present is specific for long-chain AHLs and could be responsible for the steep drop in OHC12-HSL concentration observed in supernatants of ATCC17978 cultures. The activity was also present in cell extracts obtained from shaken cultures, indicating that high AHL concentration is not required for the expression of QQ genes. The analysis of QQ activity in the supernatants of ATCC17978 cultures revealed QQ activity against long-chain AHL with a time-dependent pattern similar to the one observed in cell extracts. QQ activity against C6-HSL was also observed in the supernatants starting at the 24-h samples (data not shown). Since the pH of supernatants reached pH values of 7.8, the experiment was repeated buffering the media at pH 6.7 to avoid the spontaneous lactonolysis of the QS signals. The QQ against C6-HSL was maintained in these buffered supernatants, demonstrating the presence of enzymatic activity (data not shown). QQ activity against long-chain AHLs was also found in the cell extracts of *A. nosocomialis* M2, correlating with the concentration of the major AHL (Figure S3). QQ activity against long-chain AHLs was also found in all the *A. baumannii* clinical isolates studied (Figure S5), despite one of them (Ab7) does not possess an AidA sequence (López et al., 2017). As observed for ATCC17978, none of the clinical isolates could eliminate C6-HSL activity (Figure S5).

**Figure 4:**
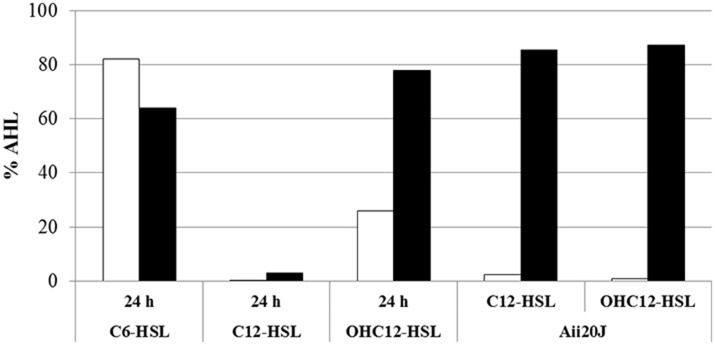
QQ activity against C6-HSL, C12, and OHC12-HSL (10 μM) in cell extracts of *A. baumannii* ATCC17978 obtained from 24 h cultures in LB. The purified Aii20J lactonase (20 μg/mL) was used as positive control. The remaining AHL concentration after 3 h of exposure to cell extracts was quantified by HPLC-MS from reaction mixtures aliquots with (black bars) or without (white bars) acidification to reverse the lactonolysis. PBS was used as negative control. Values reported are normalized to the percentage of AHL retrieved from PBS reaction mixtures incubated the same way.

In order to further confirm that the QQ activity detected in *A. baumannii* ATCC17978 is enzymatic and preferentially degrades long-chain AHLs, the signals C6, C12, and OHC12-HSL were exposed to a cell extract from a 24 h culture of ATCC17978 for 3 h and the remaining AHL concentration was quantified using HPLC-MS. ATCC17978 extracts completely degraded C12-HSL and around 75% of OHC12-HSL in 3 h, confirming the QQ enzymatic activity in *A. baumannii* ATCC17978 (Figure 4). On the contrary, the activity against C6-HSL in the extracts was very low. Additionally, aliquots of the reaction mixtures were acidified to pH 2 to facilitate the recircularization of the lactonized homoserine ring in degraded AHLs (Yates et al., 2002). If a lactonase is responsible for the QQ enzymatic activity against AHLs, an increase in the concentration of AHL after acidification would be expected, as shown for the purified lactonase Aii20J (Figure 4). In *A. baumannii* ATCC17978, the recovery after the acidification of the reaction mixtures could only be obtained for OHC12-HSL (Figure 4), confirming the presence of an AHL-lactonase in the extracts. On the contrary, the acidification did not allow the recovery of C12-HSL, indicating the possible existence of several QQ enzymes with distinct AHL-degrading activity in ATCC17978, which could explain the differences in recovery between both signals.

### Identification and cloning of putative QQ sequences in *A. baumannii* ATCC17978

The results obtained from the acidification assays, together with the fact that QQ activity was found in the clinical isolate *A. baumannii* Ab7 that does not possess an AidA sequence in its genome, prompted us to search the genome of *A. baumannii* ATCC17978 for additional QQ sequences. The genome was searched using a collection of QQ sequences including both, acylases and lactonases, with demonstrated or putative activity (Romero et al., 2015; Muras et al., 2018a). The search specifically included the acylase AmiE described in *Acinetobacter* sp. Ooi24 (Ochiai et al., 2014), the lactonase AidE from *Acinetobacter* sp. 77 and the putative lactonases YtnP from *A. baumannii* A155, belonging to the metallo-β-lactamases family and containing the conserved domain HXHXDH (Arivett et al., 2015; Liu et al., 2017) and Y2-AiiA, that presents an aspartate instead of the second histidine in the conserved domain (HXDXDH) (Arivett et al., 2015). No putative acylase sequence was found in the genome of ATCC17978. On the contrary, besides de α/β hydrolase AidA (A1S_1757), seven sequences of putative lactonases were found, sharing ID percentages between 23 and 30% at aminoacidic level with the sequence of the AiiA lactonase of *Bacillus* sp. 240B1 (Dong et al., 2000; Table S3). The sequence A1S_2662 corresponds to the putative lactonase YtnP from *A. baumannii* strain A155 deposited in NCBI database (ID 99%, cover 100%), while the sequence A1S_2864 corresponds to the putative lactonase Y2-AiiA from the same strain (Arivett et al., 2015; Table S3). These two sequences are present in several *Acinetobacter* strains (Vallenet et al., 2008; Kang and Park, 2010; Figure S6) although the QQ activity has not been proved in any of them. The remaining 5 sequences presented the zinc-binding domain characteristic of the superfamily of metallo-β-lactamases (Bebrone, 2007). *A. nosocomialis* M2 does not possess a gene identical to AidA, but an α/β-hydrolase sharing a 35% of identity with AidA was found (Access number WP_022575648.1). The 7 lactonase sequences found in ATCC17978 could be also found in the genome of *A. nosocomialis* M2, sharing % ID higher than 95% in five cases: A1S_2270, A1S_1708, A1S_2194, A1S_2864, and A1S_2662. Lower ID percentages were found in M2 sequences for two of the enzymes with demonstrated activity: A1S_1876 (ID 29%, cover 31%) and A1S_0383 (ID 34%, cover 85%). All lactonase sequences could be amplified in the *A. baumannii* clinical isolates except for A1S_2194, probably due to the PCR conditions. The conservation of these sequences within and between species of *Acinetobacter* indicates a relevant physiological role of these enzymes.

### Cloning and over-expression of the putative QQ enzymes in *E. coli*

In order to certify the QQ activity of the putative lactonases found in the genome of ATCC17978, we attempted to amplify and subclone in *E. coli* the 6 sequences presenting the highest identity to the *Bacillus* enzyme AiiA, A1S_2270 was dismissed due to the low cover percentage of identity of the sequence, although presenting the typical conserved domain (Table S3). AidA was also subcloned in *E. coli* in order to compare the specificity of the different QQ enzymes. Only 5 of the 6 selected genes could be amplified by PCR using specific primers. Sequence A1S_2194 could not be amplified even when the annealing temperature was lowered. The amplified sequences were subcloned in pET28c(+) in order to add a poly-Histidine tag to the sequence to facilitate the purification of the transgenic enzyme. Finally, recombinant *E. coli* colonies could be obtained for 3 lactonase sequences: A1S_2662 (YtnP), A1S_0383, and A1S_1876 as well as for the already described α/β hydrolase AidA. After checking the correct size and sequence of the inserts, the 4 enzymes were over-expressed and purified. The 4 enzymes were produced as soluble protein in *E. coli* BL21(DE3)plysS with the expected molecular weight taking into account the addition of the poly-His tag: ~25.88 kDa for A1S_0383, ~35.17 KDa for A1S_1876, ~37.60 kDa for A1S_2662 (YtnP) and ~33.3 kDa for AidA (Figure S7).

**Figure 5:**
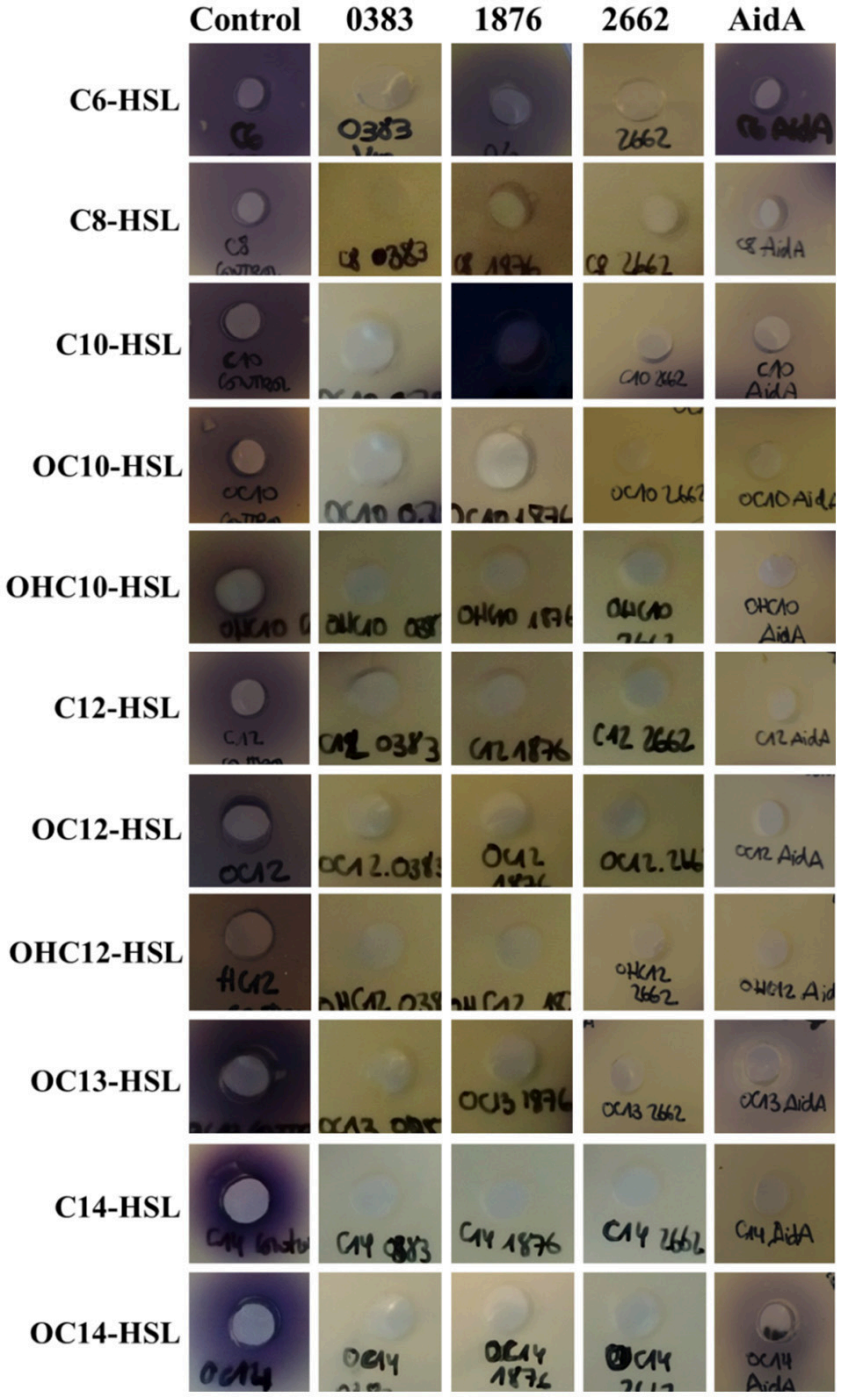
Substrate specificity of the purified lactonases A1S_0383, A1S_1876, A1S_2662, and AidA from *A. baumannii* ATCC17978. Purified lactonases were mixed at 10xMAC concentration with AHLs (10 μM) for 24 h and the remaining AHL was evaluated in bioassays with *C. violaceum* CV026 or VIR07 biosensors. Each signal in PBS pH 6.7 was used as negative control.

The 4 purified enzymes showed quorum quenching activity *in vitro*, although important differences were found regarding substrate specificity. Two of the enzymes, A1S_0383 and A1S_2662 were able to degrade all the AHLs tested, while A1S_1876 and AidA were not able to degrade the short-chain AHL C6-HSL (Figure 5). The minimum active concentration, defined as the amount of enzyme required to fully eliminate the activity of a 10 μM solution of C12-HSL in 24 h, as detected by the *C. violaceum* plate assay, was also very different among the different enzymes: 15 μg/mL for A1S_0383, 0.3 μg/mL for A1S_1876, 1.7 μg/mL for A1S_2662, and 0.8 μg/mL for AidA (data not shown).

### Expression of QQ sequences in *A. baumannii* ATCC17978

**Figure 6:**
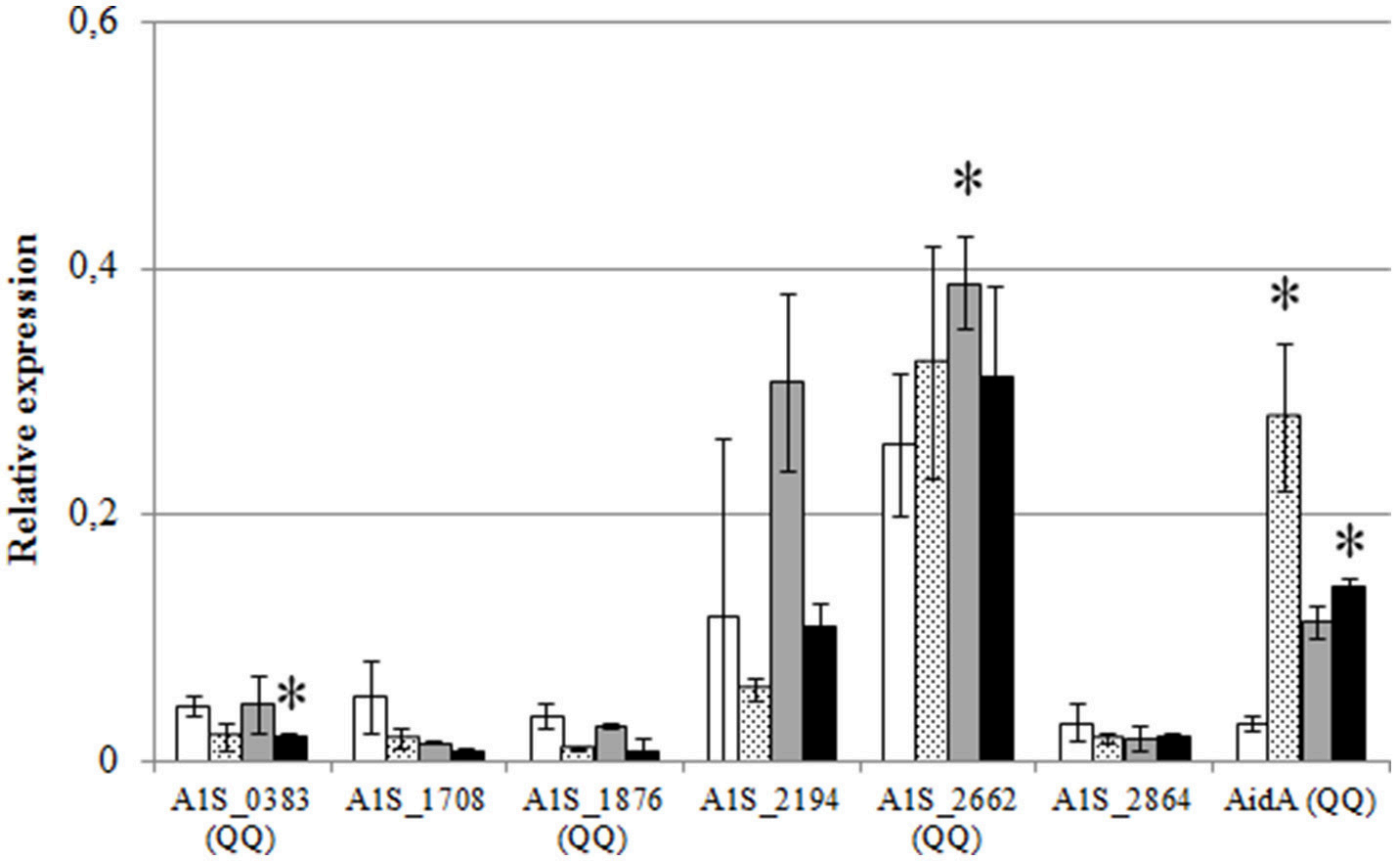
Relative expression of the QQ lactonase genes and the α/β hydrolase AidA gene from *A. baumannii* ATCC17978, in shaken LB (white bars), static LB (dotted bars), shaken low-salt LB (gray bars), or static low-salt LB (black bars). Gene expression was normalized related to the *rpoB* gene. Error bars represent the standard deviations. Asterisks indicate statistically significant changes (Student's *t*-test, *p* < 0.05) with respect to the static LB condition.

In order to evaluate if the expression of the identified lactonases was actively regulated in *A. baumannii*, a qPCR analysis was carried out under different culture conditions. The RNA was extracted after 6 h from shaken and static cultures in LB and LS-LB (Figure 6) and the expression of AidA and the additional 6 lactonase sequences found in the genome was analyzed. No significant change in expression of the 6 new lactonases was observed between static and shaken conditions, but AidA was significantly over-expressed in static conditions in LB medium (Figure 6) suggesting an activation of this enzyme as a consequence of the activation of the QS system. The wide-spectrum lactonase A1S_2662 was expressed at high levels in all conditions, but as for A1S_1876 and the putative lactonases A1S_0383, A1S_2194, the expression only increased in LS-LB under shaken conditions, indicating that the expression of these two genes is not under the control of the QS system.

**Figure 7:**
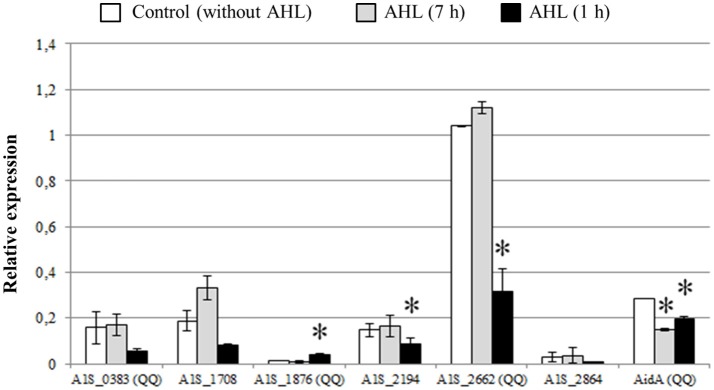
Relative expression of QQ lactonase genes and α/β hydrolase AidA gene from *A. baumannii* ATCC17978 in response to OHC12-HSL. The AHL was added at 0 h (for 7 h) or 6 h (for 1 h) of culture growth. Gene expression was normalized related to the *rpoB* gene. Error bars represent the standard deviations. Asterisks indicate statistically significant changes (Student's *t*-test, *p* < 0.05) with respect to the static LB condition.

In order to further evaluate if the expression of the identified lactonases was activated by the presence of AHLs, we added OHC12-HSL, the major AHL found in *A. baumannii* ATCC17978, either from the beginning of the cultures or after 6 h (Figure 7). When the AHL was added at the beginning of the cultures no significant change in the expression of the lactonases was found, except for AidA that suffered a slight but significant decrease in its expression. On the contrary, the addition of the AHL to 6-h cultures caused a rapid decrease in the expression of all lactonases, except for A1S_1876, a lactonase with a low basal level of expression, that suffered a three-fold increase after the addition of OHC12-HSL (Figure 7).

### Effect of exogenous quorum quenching on motility and biofilm formation

As a first approach to assess the importance of the QS system in the control of the expression of virulence factors in *A. baumannii* ATCC17978, the wide spectrum QQ enzyme Aii20J (Mayer et al., 2015) was used to try to block surface-associated motility and biofilm formation, two phenotypes previously described as being QS-controlled in different *Acinetobacter* spp. Previously, the capacity of Aii20J to effectively eliminate OHC12-HSL, the major AHL present in ATCC17978, was confirmed (Figure 4).

**Figure 8:**
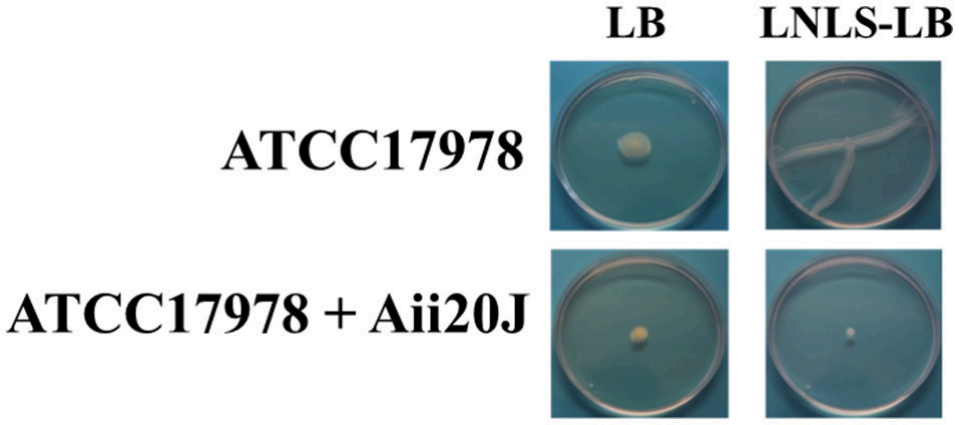
Surface-motility assay of *A. baumannii* ATCC17978, with or without the addition of the QQ enzyme Aii20J (20 μg/mL). Cells were inoculated on LB or LNLS-LB 0.25% Eiken agar plates. Surface-associated motility was inspected after 14 h of incubation at 37°C. Images are representative results of 3 independent experiments.

Surface-associated motility in *A. baumannii* ATCC17978 could only be observed in LNLS-LB medium in Eiken agar (Figure 8), presenting a characteristic tentacle-like pattern. The addition of the QQ enzyme Aii20J to the inoculum of the plates was enough to completely block the motility in these conditions. On the contrary, *A. nosocomialis* M2 presented a hyper-motile phenotype in LB medium at 37°C, being able to cover the whole plate in 14 h. This phenotype changed to a tentacle-like phenotype in the LNLS-LB medium (Figure S8). In the case of *A. nosocomialis* M2, the QQ enzyme Aii20J was not able to counteract the motility phenotype in any of the culture media tested, indicating important differences in the role of the QS system in the motility among *Acinetobacter* spp.

**Figure 9:**
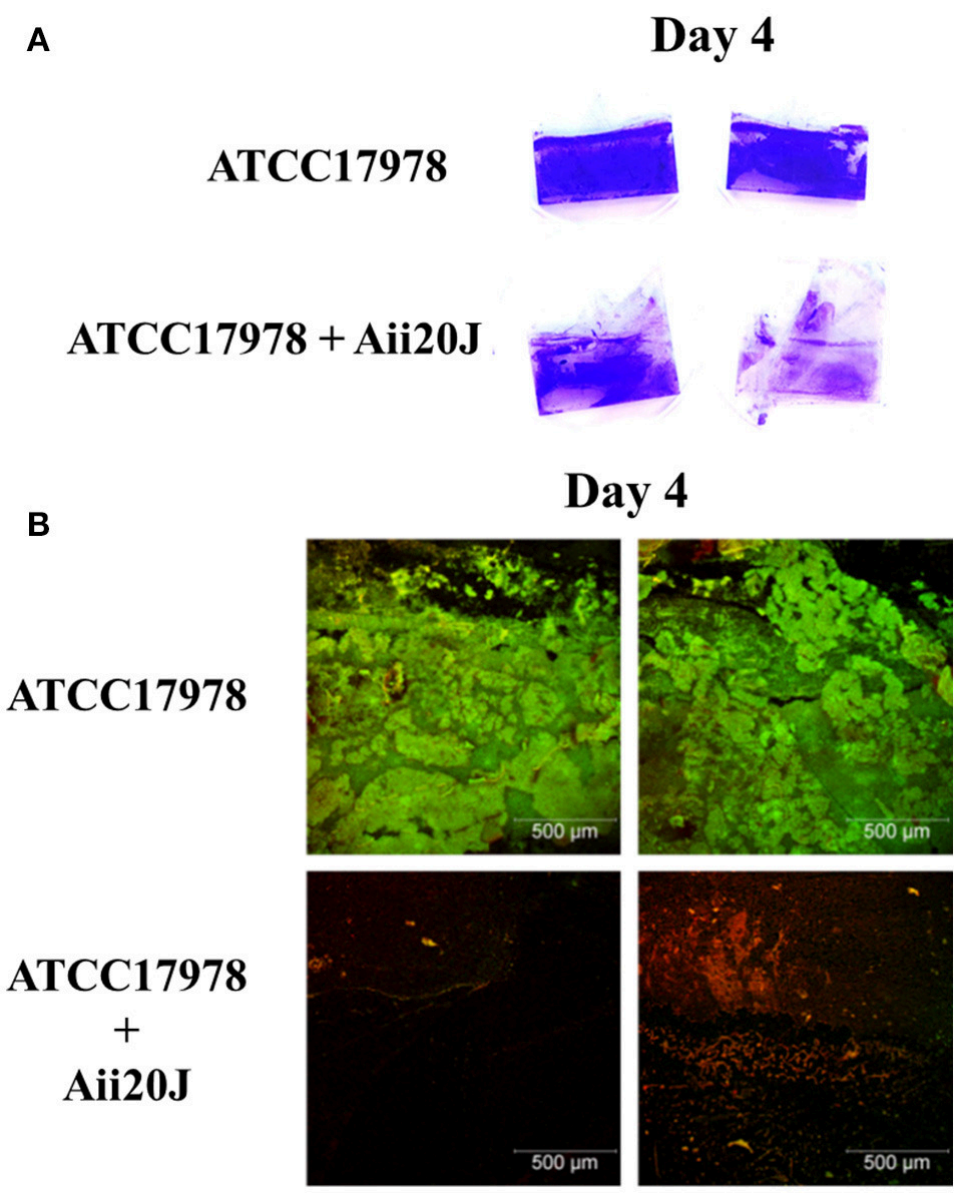
Biofilm formation of *A. baumannii* ATCC17978 in the Amsterdam active attachment model. After 4 days of growth in low-salt LB medium, with or without QQ enzyme Aii20J (20 μg/mL), biofilm was stained with **(A)** crystal violet or **(B)** SYTO9^TM^ and propidium iodide to distinguish live cells from dead cells. Images corresponding with the biofilm formed close to the interface liquid-air are representative of several replicates.

Despite differences in the structure of the biofilm formed in the presence of the QQ enzyme could be observed macroscopically (Figure 9A), the quantification of crystal violet staining could not detect significant differences in biofilm formation in *A. baumannii* ATCC17978 between the control and the Aii20J-treated cultures (data not shown). On the contrary, confocal microscopy observations revealed important differences: while a continuous biofilm of live bacteria could be observed in the control cultures, mainly close to the interface liquid-air, the coverslips incubated in the presence of Aii20J showed almost no presence of attached live cells (Figure 9B). The OD of the culture media was equal between control and QQ-treated cultures during the 4 days of incubation, and therefore the observed differences are not derived from growth inhibition (data not shown).

## Discussion

Our results strongly indicate that AHL production in *A. baumannii* ATCC17978 and *A. nosocomialis* M2 is up-regulated only when grown in static culture conditions, since no AHLs could be detected in the supernatants from shaken cultures even in 100-fold-concentrated extracts. The small differences in growth obtained between shaken and static cultures do not justify this dramatic change in AHL production (Figure S1). Moreover, none of 7 clinical *A. baumannii* isolates produced AHLs in shaken cultures, while significant amounts of AHLs could be found in the culture media of all of them in static conditions. In many cases, the identification of the AHLs produced by different *Acinetobacter* spp. was only possible after the over-expression of the AbaI synthase in *E. coli* (Niu et al., 2008; Chan et al., 2014). Niu et al. (2008) could obtain a dim response of the *A. tumefaciens* biosensor in 8,000- to 10,000-fold concentrated extracts of the culture medium in *A. nosocomialis* M2, although the culture conditions (shaken/not shaken) were not specified. Since AbaI is still expressed at low level in shaken cultures (Figure 3), we cannot completely disregard that a very small amount of AHLs is still produced in shaken cultures, although the biological significance of such low concentration of QS signals could be considered neglectable in comparison with the concentrations achieved in static cultures. The need of static culture conditions for AHL production could also explain the low percentages of AHL-producing strains found among 55 *Acinetobacter* clinical isolates (Anbazhagan et al., 2012). The absence of AHL signals in shaken cultures seems to be the result of the low expression of the AHL synthase AbaI, that is clearly up-regulated in static conditions, and not derived from a fast turn-over of the signals. These results indicate that the activation of the AHL-mediated QS system could be dependent on surface or cell-to-cell attachment in *Acinetobacter* spp. The inactivation of the QS system in shaken cultures is coherent with a previous observation reporting that pellicle formation or motility, two traits that are QS-dependent in *Acinetobacter* spp., were impaired when *A. baumannii* ATCC17978 was pre-incubated under shaking conditions. On the contrary, un-shaken cultures produced both QS dependent phenotypes (Chen et al., 2017).

To our knowledge, this is the first time that the requirement of static conditions for the activation of QS system is described. The need of mechanical cues for the detection of host cell surfaces and the expression of virulence factors has been already described in *E. coli* O157:H7 (Alsharif et al., 2015). A similar requirement was also observed in *Pseudomonas aeruginosa* that needs both, surface attachment and an active QS system for the expression of virulence genes (Siryaporn et al., 2014). In *P. aeruginosa* the chemotaxis-like chemosensor system Chp regulates the Type IV pili, that act as surface mechanosensors, and also regulates cAMP, that acts as a messenger through the virulence factor regulator (Vfr) to activate the QS circuits (Persat et al., 2015). In *A. baumannii* cAMP has been suggested to act as a regulator of the QS operon (Giles et al., 2015), and recently the two-component system CheA/Y (A1S_2811), homologous to Chp system in *P. aeruginosa* (Whitchurch et al., 2004) has been described (Chen et al., 2017). Indeed, the mutation of CheA in ATCC17978 results in a lower transcription of *abaI* and the *csu* operon and in the inhibition of motility and pellicle formation. The addition of C10-HSL restores these activities in the mutant, indicating that CheA may be controlling the expression of the motility-related *csu* operon through the expression of the QS operon (Chen et al., 2017). The *csu* operon is necessary for type I pili formation, cell attachment to plastic surfaces and latter formation of biofilms by *A. baumannii* and is thought to be under the control of the QS system (Tomaras et al., 2003; Clemmer et al., 2011; Eijkelkamp et al., 2011; Luo et al., 2015). It has been previously described that QS and *csu* operons are over-expressed in biofilms in comparison with planktonic cultures of *A. baumannii* ATCC17978 (Rumbo-Feal et al., 2013). These observations together with the fact that both, *abaI* and *csuD* are up-regulated under the static conditions required to trigger the presence of AHLs in the culture media (Figure 3), strongly support the possibility that CheA constitutes the attachment-dependent master regulator of the QS operon in *A. baumannii* ATCC17978, an hypothesis that should be further explored.

HPLC-MS analysis of the QS signals produced by *A. baumannii* ATCC17978 and *A. nosocomialis* M2 in static conditions revealed a similar complex AHL profile in both species, with OHC12-HSL as the major AHL, reaching a concentration around 1–2 orders of magnitude higher than the secondary AHLs. Only small differences in AHL profile are found between the two species that presented a more complex AHL profile in the rich LB medium than in the low-nutrient-low salt medium. Several studies have reported variations in the QS signals produced by *Acinetobacter* spp. using different species and media, however in most cases more than one AHL was reported (González et al., 2001, 2009; Sarkar and Chakraborty, 2007; Kang and Park, 2010; Chan et al., 2011, 2014; Bitrian et al., 2012; Kim and Park, 2013; How et al., 2015). A wide range of intraspecific variability was also found in the AHL profile within *A. baumannii* clinical isolates, since OHC12-HSL was present in only 4 of 7 strains when cultured in the rich LB medium. Intraspecific variation in AHL profile has been also reported in other important pathogens, such as *Serratia liquefaciens* (Remuzgo-Martínez et al., 2015). In the case of *A. baumannii*, these differences could be derived from differences in the control of the expression of the *abaI* synthase that is present in all isolates, or in the QQ activity, that was also present in all of them. Decreasing the salinity of the culture medium produced a five-fold increase in the concentration of the major AHL in *A. baumannii* ATCC17978, although no changes in growth rate were observed. This increase in signal concentration also seems to be derived from an increase in the expression of *abaI* (Figure 3A). Low salinity also activates the expression of *csuD*, which is related to surface-associated motility, a trait that has been reported to be negatively affected by high salinity values in *A. baumannii* ATCC17978 (Pour et al., 2011; McQueary et al., 2012). It is therefore plausible that additional regulators are interacting downstream the hypothetical surface-attachment sensor in order to fine-tune signal production. The complexity of this signal network that changes depending on culture conditions indicates the existence of an intricate net of signals that integrate information from several physicochemical and nutritional factors. A single channel which specifically discriminates between the presence of single and multiple autoinducers, leading to synergistic responses has been described before in *Vibrio harveyi* (Mok et al., 2003). In *P. aeruginosa*, nutritional and environmental signals selectively affect the different QS systems (Welsh and Blackwell, 2016). Therefore, it appears likely that a similar mechanism could be present in other bacteria producing different signals under different environmental stimuli.

The sharp decrease in AHL concentration upon stationary-phase achievement that correlates with the appearance of QQ activity in the cell extracts strongly indicates an active endogenous regulation of the AHL signals through enzymatic degradation. A rapid turnover of OC12-HSL was also observed in the QQ-active *Acinetobacter* sp. isolate GG2 (Chan et al., 2011). The self-regulation of AHL concentration has been already reported in *Agrobacterium tumefaciens* that activates the lactonase AttM to degrade its own signal as a response to starvation signals (Zhang et al., 2002; Uroz et al., 2009). A marine strain of *Shewanella* also degrades its own AHLs during stationary phase through a lactonase and acylase/amidase activities (Tait et al., 2009). Recently, a novel QQ enzyme, the α/β hydrolase AidA that is over-expressed in response to the non-cognate OC12-HSL has been identified in several clinical isolates of *A. baumannii* (López et al., 2017). AidA is also present in *A. baumannii* ATCC17978 and our results confirm that it is up-regulated in early-stages of the AHL-producing static cultures (Figure 6). It should be noted that AidA expression is lower under low-salt conditions that results in a higher OHC12-HSL concentration, which may indicate a direct involvement of AidA in signal degradation. Nevertheless, since AidA is not present in *A. noscomialis* M2 that presents the same pattern of AHL self-degradation, it is possible that several enzymes are active in the self-degradation process. Importantly, the addition of the cognate OHC12-HSL did not cause any significant change in AidA expression (Figure 7). It is therefore plausible that AidA is not under the direct control of the QS operon, but its expression may be controlled by additional environmental cues.

A surprisingly high number of lactonase sequences was found in the genome of *A. baumannii* ATCC17978, all of them belonging to the metallo-β-lactamase family, although only 3 of them could be subcloned in *E. coli* to confirm its AHL-lactonase activity. The presence of lactonase-like activity has been reported in other members of the genus *Acinetobacter* isolated from plant rhizosphere (Kang et al., 2004; Chan et al., 2011) that degrade both, long and short chain AHLs, and can be recovered after acidification (Chan et al., 2011). Multiple QQ enzymes have been found in *P. aeruginosa* (Huang et al., 2006) as well as in *Deinococcus radiodurans, Hyphomonas neptunium, Photorhabdus luminescens*, and *Rhizobium* sp. (Kalia et al., 2011; Krysciak et al., 2011). In *Rhizobium* sp. up to 5 QQ enzymes, including the two lactonases DhlR and QsdR1 have been described, although the involvement on the self-control of the AHL signals could not be demonstrated (Krysciak et al., 2011). The presence of 5 of the 6 lactonase sequences could be confirmed in all the clinical isolates as well as in *A. nosocomialis* M2. Among the 3 lactonases that could be subcloned and purified to prove its QQ activity, the lactonase A1S_2662, that corresponds to the putative lactonase YtnP found in the genome of *A. baumannii* strain A155 (Arivett et al., 2015) and is also highly conserved in *A. nosocomialis* M2, is expressed at high levels in all culture conditions (Figure 6). The only lactonase that is clearly activated by the addition of the cognate OHC12-HSL is A1S_1876, while all the others are down-regulated or unaffected (Figure 7), being therefore a good candidate to be under the control of the QS system. A1S_1876 is transcribed at low levels in early log-phase static cultures (Figure 6), but it should be noted that the transgenic enzyme presents the highest specific activity among the 4 enzymes that have been subcloned and purified.

Due to lack of recovery observed after acidification of C12-HSL treated with *A. baumannii* cell extracts (Figure 4), we cannot completely exclude the presence of additional acylase-type QQ enzymes in this species. Enzymatic activity against long chain AHLs had been identified previously in the wastewater isolate *Acinetobacter* sp. Ooi24 and the QQ enzyme responsible was identified as an AHL-acylase (Ochiai et al., 2014). The lack of conserved domains in AHL-acylases difficult the identification of the sequences in the bacterial genomes, and therefore, the number of QQ enzymes present in *A. baumannii* could be even greater than described here. Despite substrate promiscuity has been already described in members of the metallo-β-lactamase family with AHL degradation capacity (Miraula et al., 2016), and therefore additional metabolic activities of the lactonases identified in *A. baumannii* cannot be completely disregarded, the redundancy and differential regulation of the QQ enzymes found in ATCC17978 provide a strong evidence of the importance of the AHL-mediated QS/QQ network in this species. Since some differences in the substrate specificity have been found among the 4 QQ enzymes that could be cloned, it is possible that *A. baumannii* uses this battery of QQ enzymes to differentially regulate the relative concentration of exogenous, short-chain AHLs and the endogenous principal long-chain AHL. Further studies are required to assess the role of the QQ activity present in *A. baumannii* strains in controlling the QS regulation cascade.

The exogenous addition of the wide-spectrum lactonase Aii20J completely blocked motility and biofilm formation in *A. baumannii* ATCC17978, confirming a key role of AHL-mediated QS in the expression of these two important virulence factors in this strain. Important intra-species variability in surface-associated motility and its response to QQ has been recently reported in clinical isolates of *A. baumannii* (López et al., 2017). *A. baumannii* ATCC17978 behaves in the same way as the clinical isolate Ab7, that does not possess an AidA sequence in its genome, and therefore the intrinsic QQ system does not seem to be involved in *in vitro* motility pattern and response to extrinsic QQ. Results indicate that the endogenous QQ activity present in this strain serves only to fine-tune the AHL production and that QQ strategies can be suitable as anti-pathogenic strategy in this species. In the case of *A. nosocomialis* M2, the mutation of the *abaI* gene resulted in a decrease in motility at 30°C, indicating that this trait is also under the control of the QS system in this strain (Clemmer et al., 2011). Although this effect was also observed in our experiments at 30°C (data not shown), the sensitivity of the strain to QQ was lost at 37°C, indicating important differences in the control of surface-associated motility between these two species. Regarding the effect of QQ on biofilm formation, the transgenic expression of the *Geobacillus kaustophilus* lactonase GKL in a clinical isolate of *A. baumannii* disrupted biofilm formation (Chow et al., 2014) and the addition of the QQ lactonase MomL also diminished, but not abolished biofilm formation in *A. baumannii* LMG 10531 as measured with the crystal violet staining method (Zhang et al., 2017). In the case of *A. baumannii* ATCC17978 the number of attached live cells decreases dramatically in the presence of the exogenous added QQ enzyme Aii20J (Figure 9). These differences were not so obvious at macroscopic level with the crystal violet staining method. A more detailed study on the effect of inactivating the QS system through both, external QQ and the generation of isogenic mutants is necessary in order to ascertain the role of the QS system in biofilm formation in this important nosocomial pathogen and to further explore the potential antipathogenic capacity of QQ strategies.

## Author contributions

CM and AM carried out the experimental work. CM, MR, and AO contributed to the design, and interpretation of data. CM and AO wrote the manuscript while MR revised the manuscript. ML and MT contributed to the interpretation of the data related to QS and provided the clinical strains of *Acinetobacter*.

### Conflict of interest statement

The authors declare that the research was conducted in the absence of any commercial or financial relationships that could be construed as a potential conflict of interest.
