# Supplementary material for: Multiple Quorum Quenching Enzymes Are Active in the Nosocomial Pathogen Acinetobacter baumannii ATCC17978
Source: Front Cell Infect Microbiol. 2018 Sep 13;8:310. doi: 10.3389/fcimb.2018.00310 (PMC6146095; doi:10.3389/fcimb.2018.00310)
Supplement: Supplementary file 1 [file Data_Sheet_1.pdf]

Supplementary material

Table 1S. AHL concentration (nM) in extracted supernatants of *A. baumannii* ATCC17978 and *A. nosocomialis* M2 in static LB, LNLS-LB, or buffered LB cultures.

	***A. baumannii* ATCC17978**					***A. nosocomialis* M2**				
	**LB Medium**					**LB Medium**				
Time (h)	**OHC10**	**OC12**	**OHC12**	**OC13**	**OHC14**	**OHC10**	**OC12**	**OHC12**	**OC13**	**OHC14**
6	-	-	-	-	0,156	-	0,265	0,084	-	-
12	0,200	-	21,716	-	0,099	0,202	-	27,144	-	-
17	2,119	-	102,675	-	0,327	0,928	-	57,550	-	-
24	3,687	0,041	89,903	-	0,109	1,180	0,063	27,143	-	-
36	0,219	-	3,189	-	0,060	-	0,028	0	-	-
48	-	-	0,501	-	0,100	-	0,095	0	-	-
	**LNLS-LB Medium**					**LNLS-LB Medium**				
	**OHC10**	**OC12**	**OHC12**	**OC13**	**OHC14**	**OHC10**	**OC12**	**OHC12**	**OC13**	**OHC14**
6	0,041	-	0,591	-	-	0,162	-	20,551	-	-
12	0,289	-	23,170	-	-	1,098	-	90,089	-	-
17	1,659	-	95,218	-	-	0,620	-	28,418	-	-
24	2,078	-	38,131	-	-	0,061	-	0,167	-	-
36	0,060	-	0	-	-	-	-	0	-	-
48	-	-	0	-	-	0,008	-	0	-	-
	0,041	-	0,591	-	-					
	**Buffered LB Medium**					**Buffered LB Medium**				
	**OHC10**	**OC12**	**OHC12**	**OC13**	**OHC14**	**OHC10**	**OC12**	**OHC12**	**OC13**	**OHC14**
6	-	-	0	-	-	-	-	0	-	-
12	-	-	1,503	-	-	-	-	0,184	-	-
17	0,411	-	47,393	-	-	-	-	0,845	-	-
24	6,575	0,061	215,634	-	-	0,061	-	2,406	-	-
36	11,753	0,0368	130,099	-	-	0,232	-	10,567	-	-
48	8,391	-	20,612	-	-	0,101	-	0	-	-

-: absence of AHL

Table 2S. AHL concentration (nM) identified in extracted supernatants of clinical isolates of *A. baumannii* ATCC17978 in static LB, or LNLS-LB 24-hours cultures.

**LB Medium**					
**Clinical isolate**	**OHC10**	**OC12**	**OHC12**	**OC14**	**OHC14**
Ab1	-	-	-	-	0,009
Ab2	-	-	-	-	0,027
Ab3	0,542	0,003	12,475	-	0,012
Ab4	0,479	-	15,324	-	0,006
Ab5	-	-	-	-	-
Ab6	0,032	-	0,918	-	0,009
Ab7	0,016	-	-	-	-

**LNLS-LB Medium**					
**Clinical isolate**	**OHC10**	**OC12**	**OHC12**	**OC14**	**OHC14**
Ab1	-	-	0,092	0,015	-
Ab2	-	-	0,036	0,016	0,019
Ab3	-	-	0,048	0,017	0,048
Ab4	-	-	0,074	0,015	0,016
Ab5	-	-	0,094	0,020	-
Ab6	-	-	0,065	0,016	-
Ab7	-	0,015	0,036	0,013	0,027

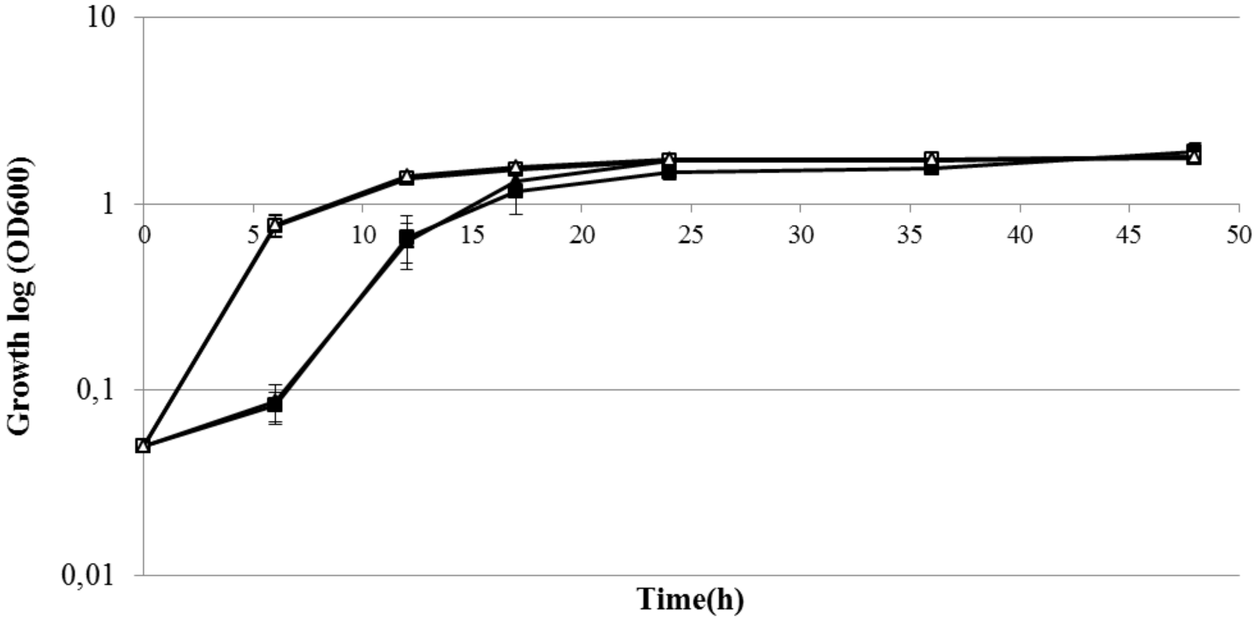

Figure 1S. Growth curves of *A. baumannii* ATCC17978 in shaken LB (white squares), static LB (filled squares), shaken low salt-LB (white triangles), or static low salt-LB (filled triangles) cultures.

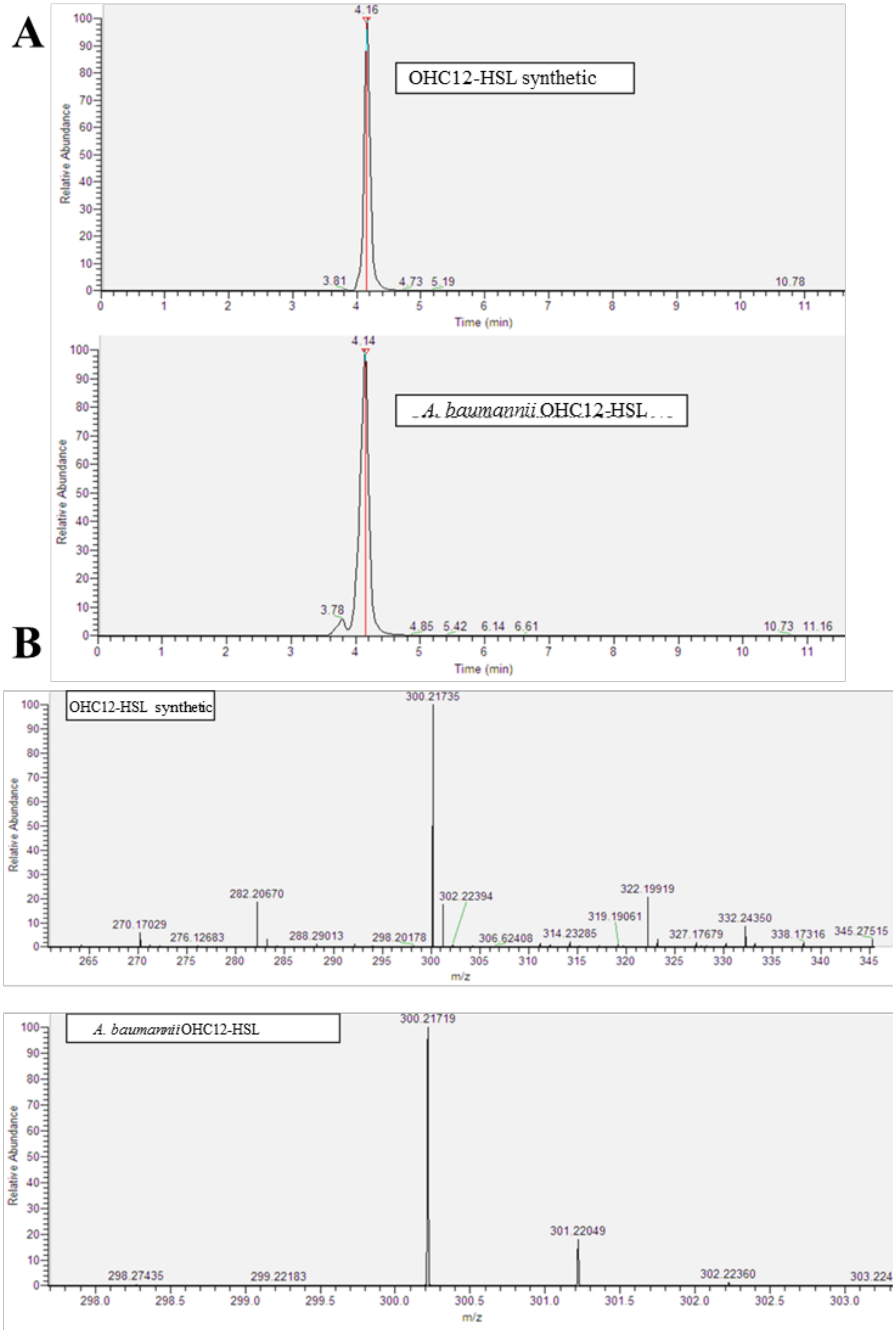

Figure 2S. A) Total ion current chromatogram from a LC-MS analysis of the synthetic OHC12-HSL synthetic (1 ppm) used as reference, and the OHC12-HSL identified in extracted supernatants of *A. baumannii* ATCC17978 in static cultures. **B)** Identification of OHC12-HSL peaks in *A. baumannii* ATCC17978 by HPLC-MS analysis.

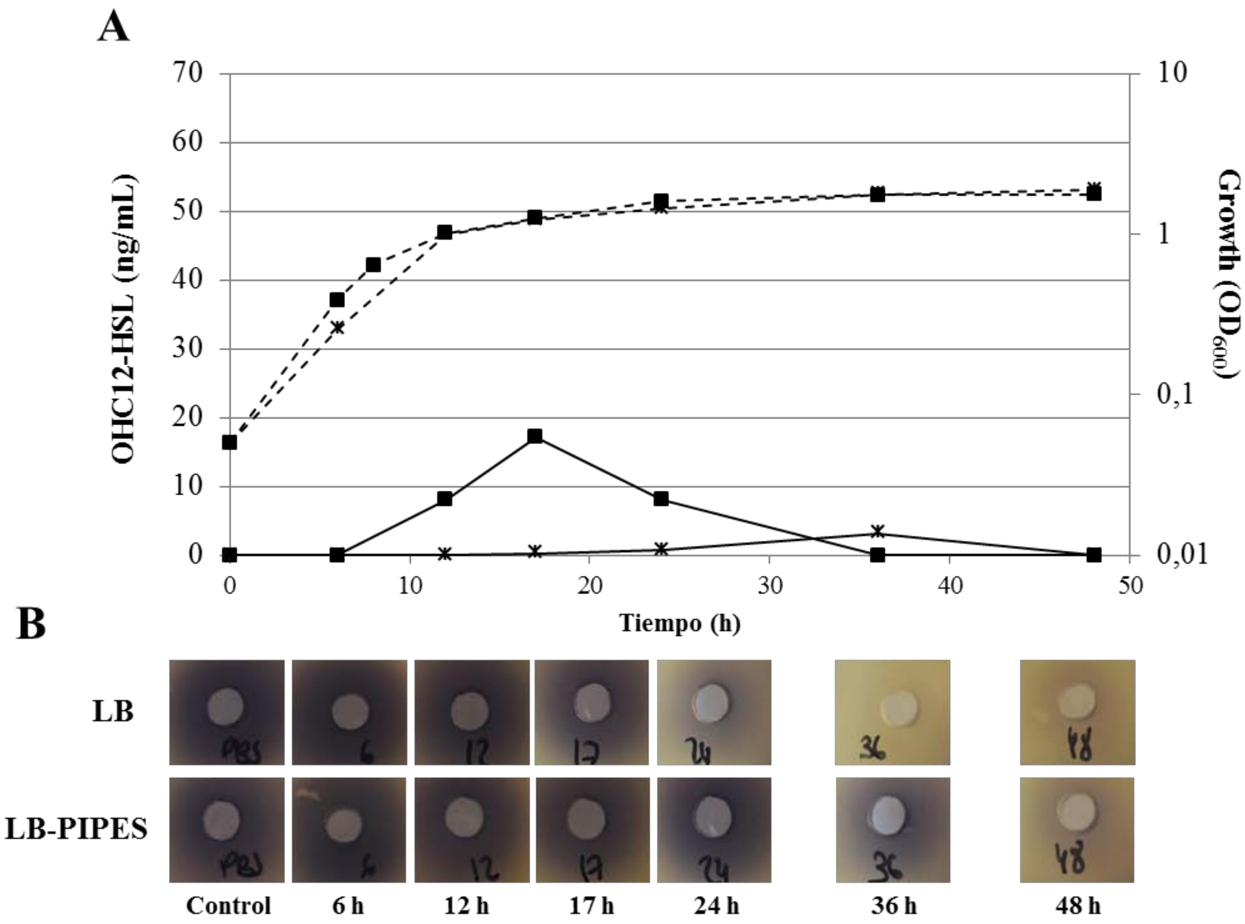

Figure 3S. A) OHC12-HSL production kinetics (continuous lines) and growth curves (discontinuous lines) in static cultures of *A. nosocomialis* M2 grown in LB (filled squares) or buffered LB (black cross) (200 mM PIPES buffer, pH 6.7) at 37ºC for 48h. **B)** Bioassay with *C. violaceum* VIR07 to detect QQ activity in cell extracts of *A. baumannii* ATCC17978. The degradation activity against exogenous C12-HSL (10 µM) was assayed in cell extracts obtained at different time points of the growth curve (6, 12, 17, 24, 36 and 48 h) under static conditions. The presence of QQ activity is revealed by the absence of violacein around the wells. PBS plus AHL samples were treated in the same way and were used as negative controls (Control).

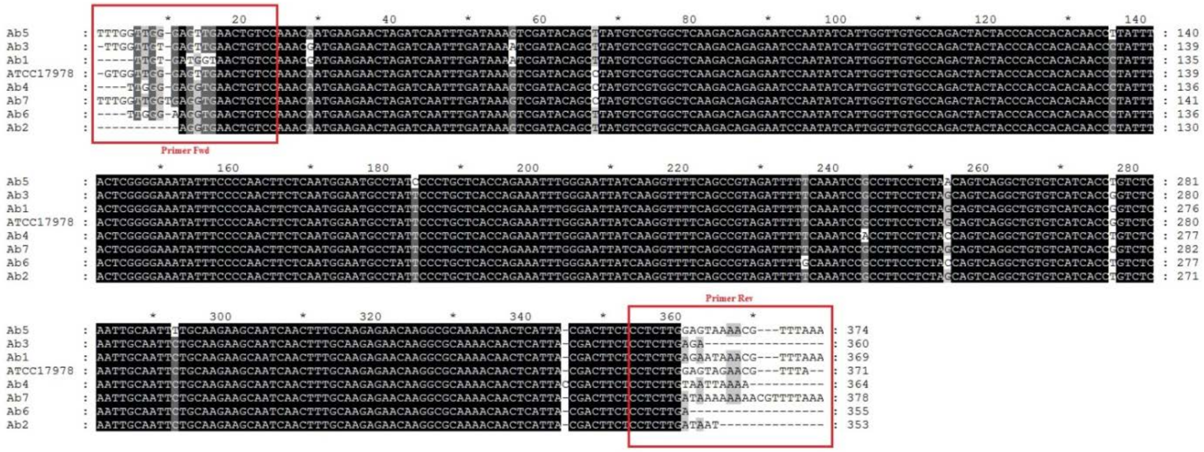

Figure 4S. Nucleotide sequence alignment of AHL-synthases from *A. baumannii* ATCC17978 and Ab1-Ab7 *A. baumannii* clinical isolates. Sequences were aligned using MUSCLE program from EMBL-EMI (https://www.ebi.ac.uk/Tools/msa/muscle/) and shaded with the Genedoc program (http://www.nrbsc.org/gfx/genedoc/). The identical and similar residues are shaded in black and grey, respectively. Primers used for PCR amplification are indicated by a red frame.

Table 3S. QQ lactonase sequences found in the genome of *A. baumannii* ATCC17978. ID and cover percentages shared with AiiA from *Bacillus* sp. 240B1 (Dong et al. 2000) and the putative lactonases YtnP and Y2-AiiA from *A. baumannii* A155 (Arivett et al. 2015) are shown. Homologous sequences to AiiA, YtnP or Y2-AiiA are shaded in grey. The sequences with QQ activity demonstrated in the present study are marked with an asterisk.

Protein	Access number	Sequence description in NCBI	ID AiiA	Cover AiiA	ID Y2-AiiA	Cover Y2-AiiA	ID YtnP	Cover YtnP
A1S_0383*	ABO10842.2	A Hypothetical protein	30	42	23	49	**24**	**71**
A1S_1708	ABO12135.1	B Beta-lactamase-like protein	23	39	26	13	43	14
A1S_1876*	ABO12303.2	C Putative metallo-beta lactamase	23	65	46	34	50	11
A1S_2194	ABO12621.2	D Putative hydroxyacylglutathione hydrolase	29	39	25	49	26	39
A1S_2270	ABO12693.2	E Metal-dependent hydrolase of the beta-lactamase superfamily III	39	9	29	24	23	35
A1S_2662*	ABO13078.2	F Putative hydrolase	**24**	**66**	**24**	**83**	**99**	**100**
A1S_2864	ABO13270.2	G Hypothetical protein	26	51	**98**	**66**	30	20

ID: sequence identity

*: QQ activity demonstrated in this study

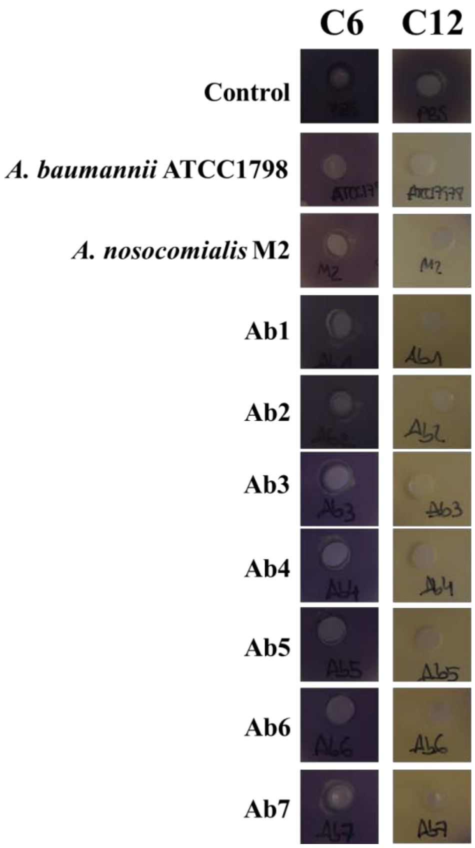

Figure 5S. Solid plate assay to detect AHL-QQ activity in live cells of 24-h cultures of *A. baumannii* ATCC17978, *A. nosocomialis* M2 and different clinical isolates from *A. baumannii*, with the AHL biosensors *C. violaceum* CV026 and VIR07. Remaining AHL activity was evaluated after 24 h of exposure of C6- or C12-HSLs 10 μM in PBS pH 6.7 to live cells. A negative control was also set in the plates with PBS pH 6.7 plus each QS signal.

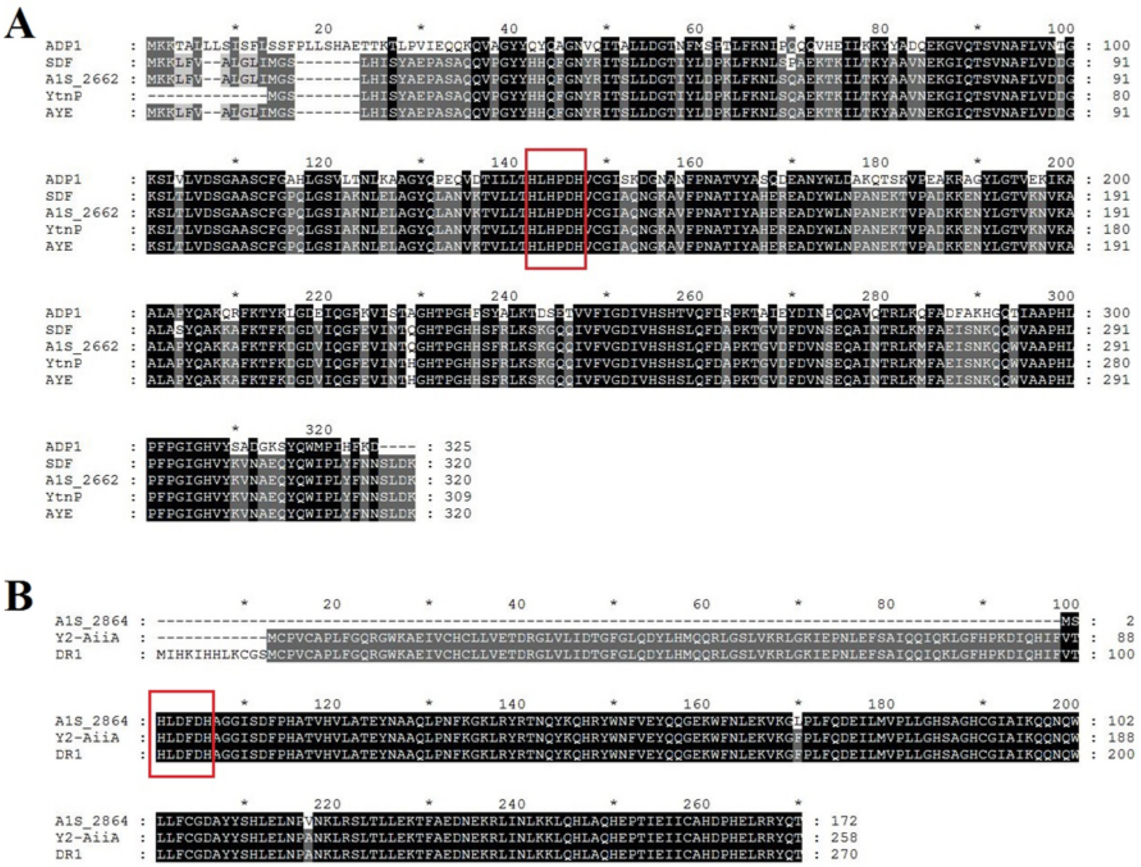

Figure 6S. Amino acid sequence alignment identified in *A. baumannii* ATCC17978 that are homologous to putative QQ sequences described in literature or deposited in NCBI. **A)** A1S_2662 from *A. baumannii* ATCC17978 and QQ putative YtnP (YtnP) from *A. baumannii* A155 (Arivett et al. 2015, access number KIQ73938.1), and AHL-lactonases from *A. baumannii* AYE (AYE), *A. baumannii* SDF (SDF) and *A. baylyi* ADP1 (ADP1) (Vallenet et al. 2008, access numbers CAM85778.1, CAP00187.1 and CAG67670.1, respectively). **B)** A1S_2864 from *A. baumannii* ATCC17978 with the putative *N*-acil homoserine lactonase Y2-AiiA de *A. baumannii* A155 (Y2-AiiA) (Arivett et al. 2015, KIQ72097.1), and putative AHL-lactonase from *Acinetobacter* sp. DR1 (Kang and Park 2010, CAM85585.1). Sequences were aligned using Clustal Omega program (https://www.ebi.ac.uk/Tools/msa/clustalo/) and were shaded with the Genedoc program (http://www.nrbsc.org/gfx/genedoc/). The identical and similar residues are shaded in black and grey, respectively. The conserved metallo-β-lactamase domains are indicated by red frames.

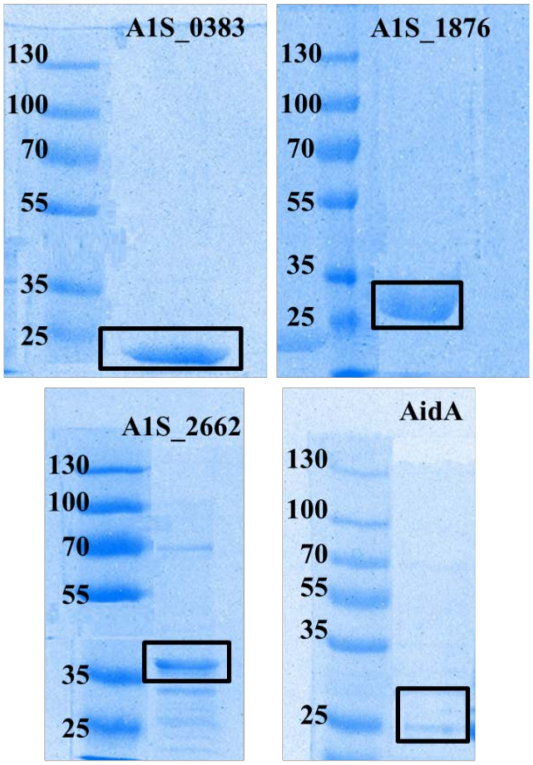

Figure 7S. SDS-PAGE gel stained with Coomasie blue of purified lactonases from *A. baumannii* ATCC17978 (full sequence, right lanes). Left lanes, protein marker (kDa). Purified proteins, including the poly-his tag and the signal peptide have the expected molecular weight.

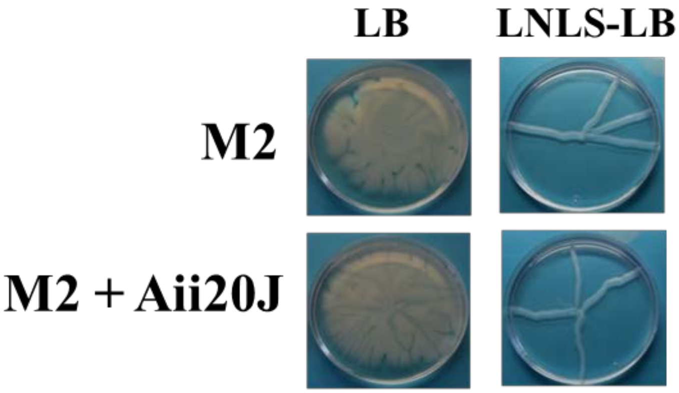

Figure 8S. Surface-motility assay of *A. nosocomialis* M2, with or without the addition of the QQ enzyme Aii20J (20 µg/mL). Cells were inoculated on LB or LNLS-LB 0.25% Eiken agar plates. Surface-associated motility was inspected after 14 h of incubation at 37ºC. Images are representative results of 3 independent experiments.
